# An exceptionally stable and widespread hydrated amorphous calcium carbonate precipitated by the dog vomit slime mold *Fuligo septica* (Myxogastria)

**DOI:** 10.1038/s41598-022-07648-9

**Published:** 2022-03-07

**Authors:** Laurence A. J. Garvie, Péter Németh, László Trif

**Affiliations:** 1 School of Earth and Space Exploration, Arizona State University, 781 East Terrace Rd., Tempe, AZ 85287-6004 USA; 2 Institute for Geological and Geochemical Research, Research Centre for Astronomy and Earth Sciences, Eötvös Loránd Research Network, Budaörsi Street 45, Budapest, 1112 Hungary; 3 Research Institute of Biomolecular and Chemical Engineering, Nanolab, Hungary, University of Pannonia, Egyetem út 10, Veszprém, 8200 Hungary; 4 Institute of Materials and Environmental Chemistry, Research Center for Natural Sciences, Magyar tudosok korutja 2, Budapest, 1117 Hungary

**Keywords:** Materials science, Mineralogy

## Abstract

Biogenic amorphous calcium carbonate (ACC) is typically metastable and can rapidly transform through aging, dehydration, and/or heating to crystalline calcium carbonate. Gaining insight into its structure and properties is typically hampered by its tendency to crystallize over short time periods once isolated from the host organism, and also by the small quantities that are usually available for study. Here we describe an exceptionally stable hydrated ACC (HACC) precipitated by the cosmopolitan slime mold *Fuligo septica* (L.) F.H. Wigg. (1780). A single slime mold can precipitate up to a gram of HACC over the course of one night. Powder x-ray diffraction (XRD) patterns, transmission electron microscopy images, infrared absorption spectra, together with the lack of optical birefringence are consistent with an amorphous material. XRD simulations, supported by thermogravimetric and evolved gas analysis data, are consistent with an intimate association of organic matter with ~ 1-nm-sized ACC units that have monohydrocalcite- and calcite-like nano-structural properties. It is postulated that this association imparts the extreme stability of the slime mold HACC by inhibiting loss of H_2_O and subsequent crystallization. The composition, structure, and thermal behavior of the HACC precipitated by *F. septica* collected over 8000 km apart and in markedly different environments, suggests a common structure, as well as similar biochemical and biomineralization mechanisms.

## Introduction

Significant effort is devoted to revealing the structure of amorphous calcium carbonate (ACC), and the mechanisms by which it forms and crystallizes^1–9^. ACC is typically a transient material that transforms into crystalline calcium carbonate. For example, laboratory synthesized ACC can transform within minutes to days into crystalline CaCO_3_^10–12^. Biogenic ACC can be broadly divided into transient anhydrous ACC and comparatively more stable material with ~ 1 mol of water^13–15^. Biogenic and synthetic hydrated ACC typically contains about 1 mol H_2_O. For example, synthetic ACC can contain between 1.13 and 1.58 mol of H_2_O^5,7,16^, with mass losses up to ~ 200 °C similar to that measured for monohydrocalcite. Though, ACC has been synthesized with lower H_2_O contents, e.g.,^5,17^. Characterization of biogenic ACC can be challenging for several reasons given that only small quantities are usually available, its presence may be masked by crystalline calcium carbonates, and because of its tendency to crystallize over short time periods once isolated from the host organism.

ACC can be stabilized in the presence of inorganic ions, including Mg^2+^ and phosphate, and organic molecules^13,14,18–20^. For example, ACC produced by cave bacteria is stable in the presence of extracellular polysaccharides^19^. While the bacteria produce a wide range of organic molecules at their cell wall surface, the presence of large amounts of long-chain fatty acids suggests that the ACC could be enclosed in micellar-like units that inhibit water infiltration, which stabilizes the ACC. Earthworms ACC can remain stable for years^21^. Its ACC contains high concentrations of amino acids, suggesting a stabilizing role of these organic compounds. Similarly, ACC from an ascidian skeleton contains glycoproteins, with high concentrations of glutamic and hydroxy amino acids: these macromolecules are suggested to play a role in the stability of the ACC^14^. The location of organic molecules, i.e., adsorbed versus bulk, can also have dramatic effects on ACC stability. For example, surface adsorption of a polymerized amino acid (poly Aspartic acid) onto ACC has a significantly higher stabilizing effect than bulk incorporation^22^. In contrast, the bulk incorporation of phosphate and hydroxyl anions into ACC improves its stability, though surface adsorption does not^22^.

Structural investigations of ACC suggest a range of short-range orderings, from vaterite-^1^, calcite-^1,3^ and monohydrocalcite-like^4^, though some studies suggest structures inconsistent with known calcium carbonate polymorphs^5^. Despite these diverse viewpoints, the structural analyses of ACC universally suggest short-range order. For example, Ca EXAFS data is consistent with a structure lacking coherence above 1.5 nm^5^, suggesting that order does not extend beyond the first coordination shell of Ca, which is about 0.4 nm from the central Ca atom^4^. Further, Sun et al.^9^ synthesized organic protected clusters of ACC with seven CaCO_3_ units with an ~ 1.4 nm core. Their ^13^C NMR and Ca K-edge EXAFS data demonstrate the rather disordered nature of the Ca sites, having a range of Ca-O coordinations, geometries, and bond lengths. However, despite this disorder, the data suggest a proto-calcite-like short-range order. In contrast, Rez et al.^8^ proposed a nanocrystallite model for ACC consisting of randomly oriented 1-nm-sized nanocrystals with water molecules between distorted nanocrystallites. Goodwin et al.^2^ developed a model for synthetic ACC consisting of a porous Ca-rich framework that supports interconnected channels containing water and carbonate molecules, though still lacking long-range order. These models and data, and those from other studies, imply that not all ACC is the same^23^, i.e., there is unlikely a single ACC structure.

A wide range of life precipitates ACC, including worms, mollusks, sponges, crustaceans, ascidia, bacteria, slime molds, avian eggshells, and plants^14,19,21,24–27^. Particularly widespread, though less frequently studied, is the structure of the calcium carbonate precipitated by the Myxogastria, a group of organisms commonly called slime molds^28–32^. Study of slime mold carbonate is typically hampered by the small sizes of most species, which produce only microscopic quantities of ACC. However, some form cm- to dm-sized spore-filled masses called aethalia, which can be covered by the peridium, which is a mineralized coating^33^. Most notable are the aethalia produced by the cosmopolitan species *Fuligo septica*^33,34^, which can be up to 75 cm across^34^. The calcium carbonate precipitated by slime molds typically occurs as micron- and submicron-sized spheres or euhedral crystals^28,29,32,33,35–37^, though rarely is the precipitated material characterized, and is variously referred to as “lime” in the literature. For example, Schoknecht^31^ employed a scanning electron microscope with x-ray microanalysis to study the calcareous deposits from a range of acellular slime molds: the presence of crystalline versus amorphous CaCO_3_ was based on crystal shapes. There is evidence to suggest that the calcium carbonate precipitated by *F. septica* in the peridium is in part amorphous^29^.

In this study, we describe and characterize the ACC formed by the cosmopolitan slime mold *F. septica* (L.) F.H. Wigg (1780)^38^, commonly known as the dog vomit or scrambled-egg slime mold. The structure, characteristics, and composition of the freshly precipitated peridium is revealed using powder x-ray diffraction (XRD), scanning and transmission electron microscopy (SEM and TEM), infrared spectroscopy (IR), thermal techniques (TG—thermogravimetric analysis, DSC—differential scanning calorimetry, and MS-EGA—mass spectroscopic evolved gas analysis), and bulk elemental analysis by proton-induced x-ray emission (PIXE) and CHN analysis. These techniques were also used to follow the laboratory dehydration of the hydrous ACC (HACC) to a dehydrated form (DACC), and the crystallization to calcite. The sequence of transformations is HACC to DACC, which is stable to 322 °C, partial crystallization of calcite between 322 and 440 °C, and complete transformation to calcite by 450 °C. A major finding of our study is that the *F. septica* HACC and DACC are indefinitely stable under normal laboratory conditions. Our data are consistent with an intimate association of organic matter with ~ 1-nm-sized ACC units that have monohydrocalcite- and calcite-like nano-structural properties. It is speculated that this association imparts the extreme stability of HACC by inhibiting the loss of H_2_O and subsequent crystallization.

## Results and discussion

### Characterization of the field-collected ACC

**Figure 1:**
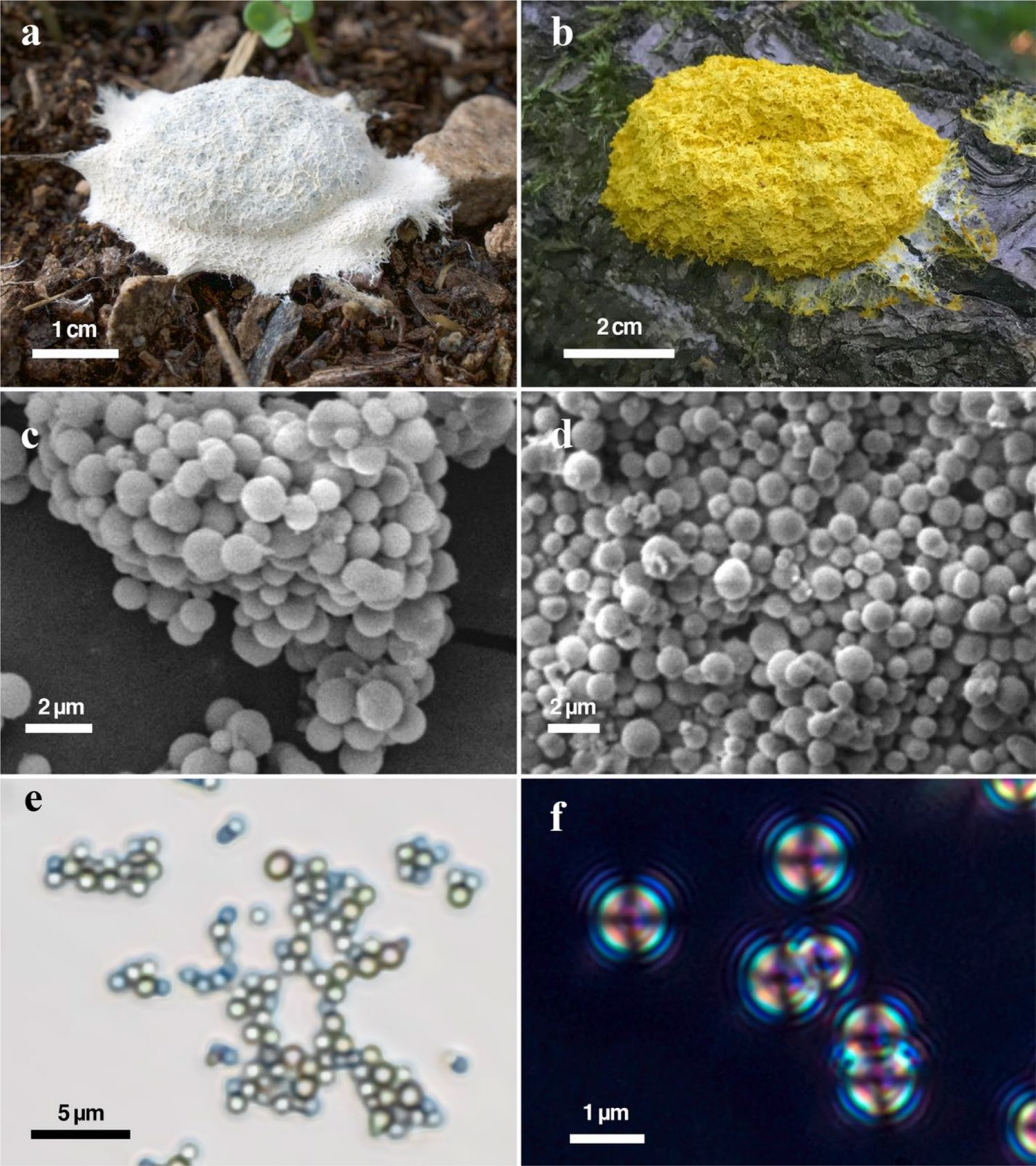
Photographs of *Fuligo septica* aethalia of (**a**) typical Arizona specimen (FSW), and (**b**) yellow specimen from the UK (FSY), and corresponding BSE SEM images of the peridium (**c**, **d**) showing the spherical morphology of the peridial HACC. (**e**) Optical transmitted-light microscope image of a cluster of HACC spheres, and (**f**) crossed-polarized transmitted-light optical image of the spheres. The spheres show dark isogyres and isochromatic rings characteristic of an amorphous material.

Aethalia from 37 *F. septica* specimens were collected (See Field Observations under “Methods” section) and stored under normal laboratory conditions. The aethalia consist of a dark purple inner spore mass and an outer light-colored peridium. The peridium is the thin, to 3-mm thick, brittle porous coating that covers and surrounds the spore-bearing aethalium (Figs. 1a, b, S1c). The specimens studied here from southern Arizona are white (referred to as FSW for **F**uligo **S**eptica **W**hite) and the UK specimens are bright canary yellow (referred to as FSY for **F**uligo **S**eptica **Y**ellow) (Figs. 1, S1, S2). The yellow of the FSY specimens is caused by a range of pigmented compounds including the tetramic acid derivative fuligorubin A^39^, which also acts as a metal chelating agent^40,41^. Observations of the Arizona specimens by the senior author showed emergence of the plasmodium during the early evening and formation of the aethalium by early morning within a few days following summer rains. By morning, the aethalia are fully developed and samples were collected for study. Fragments of the peridia free of the spores were separated for analysis (Fig. S2).

A combination of PIXE and CHN analyses were used to analyze the elemental composition of the FSY and FSW peridia (Tables 1, S1, S2). Calcium is the major cation. The FSY samples contain ~ 3.3 wt% Mn, whereas the FSW samples contain between 937 and 2186 ppm Mn. Magnesium was typically at or below the PIXE detection limit of ~ 200 ppm: the only other elements consistently above the limit of detection for the PIXE are P, S, Cl, and K (Table S1). Phosphorous content is variable and ranges from 1237 to 2850 ppm (Table S1). Carbon, H, and N are present in the as-collected, but dry peridia in the 12.15 to 14.63, 2.35 to 2.62, and 0.87 to 1.50 wt% ranges, respectively (Tables 1, S2).

**Table 1:** Major element compositions (wt%) for two FSW and one FSY HACC samples, and for monohydrocalcite.

	Major element (wt%)						
	Ca_P_	M_tot-TG_	Mn_P_	O_dif_	C	H	N
FSW21	30.5 (n = 3)	31.9	0.12	53.375	12.49	2.505	1.01
FSW22	32.4 (n = 3)	n.d	0.12	52.115	12.15	2.350	0.87
FSW_avg_	31.5 (31.5 ± 1.5)	–	0.12	52.745	12.32	2.428	0.94
FSY1	28.1 (n = 2)	31.92	3.33 (n = 2)	49.84	14.63	2.62	1.50
Monohydrocalcite	33.94	–	–	54.19	10.17	1.71	-

Additional compositional details are in Tables S1 and S2.

P—PIXE. M_tot-TG_—total metal wt% determined by TG assuming that the product after heating to 1000 °C is (Ca, Mn)O only. O_diff_—oxygen determined by the difference of the sum of the measured elements. C, H, N by bulk CHN analysis. n.d.—not determined.

**Figure 2:**
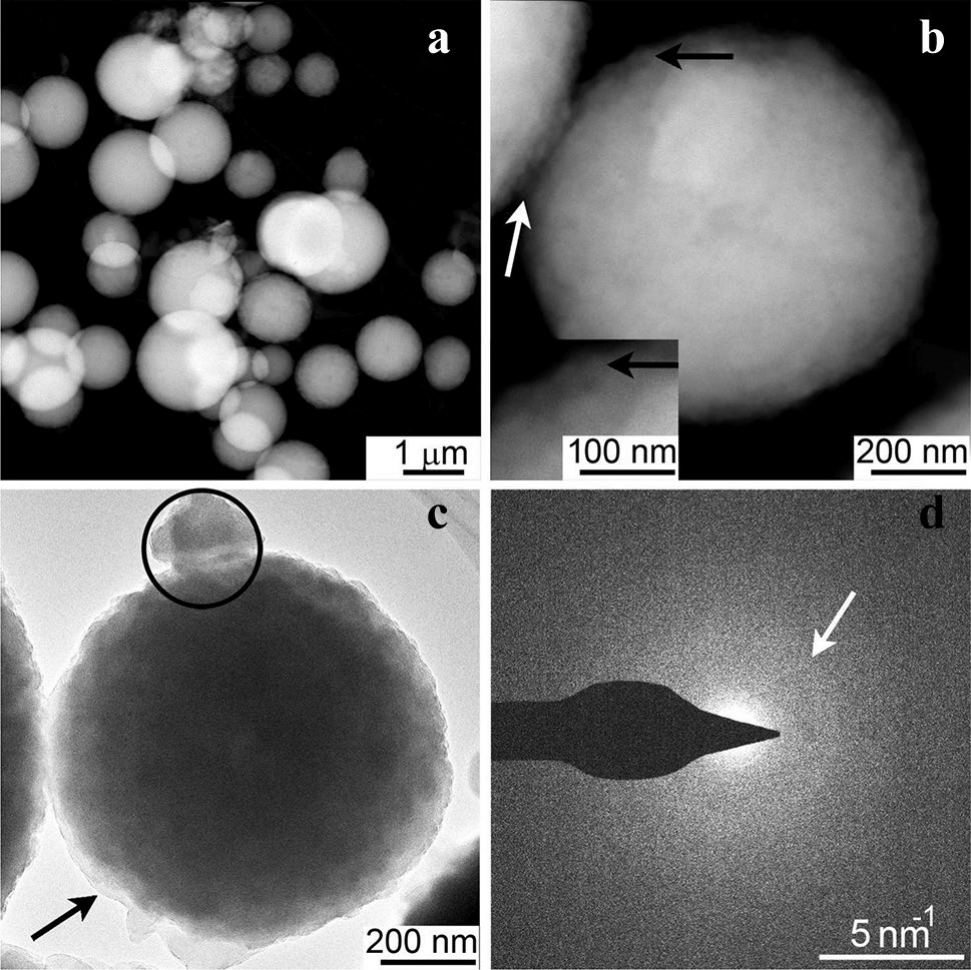
TEM images showing the morphology and structure of the FSY HACC precipitated by *Fuligo septica*. (**a**) Low-magnification HAADF-STEM image of the peridial HACC spheres. (**b**) HAADF-STEM and (**c**) BFTEM images of one sphere. Black arrow marks ~ 30 to 40-nm size quasi-spherical features. Imaged processed to more clearly reveal the internal clumping is shown in Fig. S3. (**d**) SAED pattern acquired from the circled area in (**c**). White arrow points to a diffuse diffraction ring with ~ 0.29 nm spacings.

Optical, SEM, and TEM images show that the peridia are dominated by 500- to 1500-nm-sized spheres (Figs. 1, 2, S1d), consistent with previous SEM images^29^. The spheres are isotropic under crossed-polarized transmitted light (Fig. 1f). The high-angle annular dark-field scanning TEM (HAADF-STEM) and bright-field TEM (BFTEM) images show that the spheres typically have an internally mottled appearance. This mottling is especially evident in the BFTEM images, which show rounded, 30- to 40-nm-sized electron dense units (Figs. 2c, S3). The selected-area electron diffraction (SAED) patterns of the spheres show diffuse rings (Fig. 2d), similar to what was reported by Enyedi et al.^19^ for bacterially precipitated ACC.

**Figure 3:**
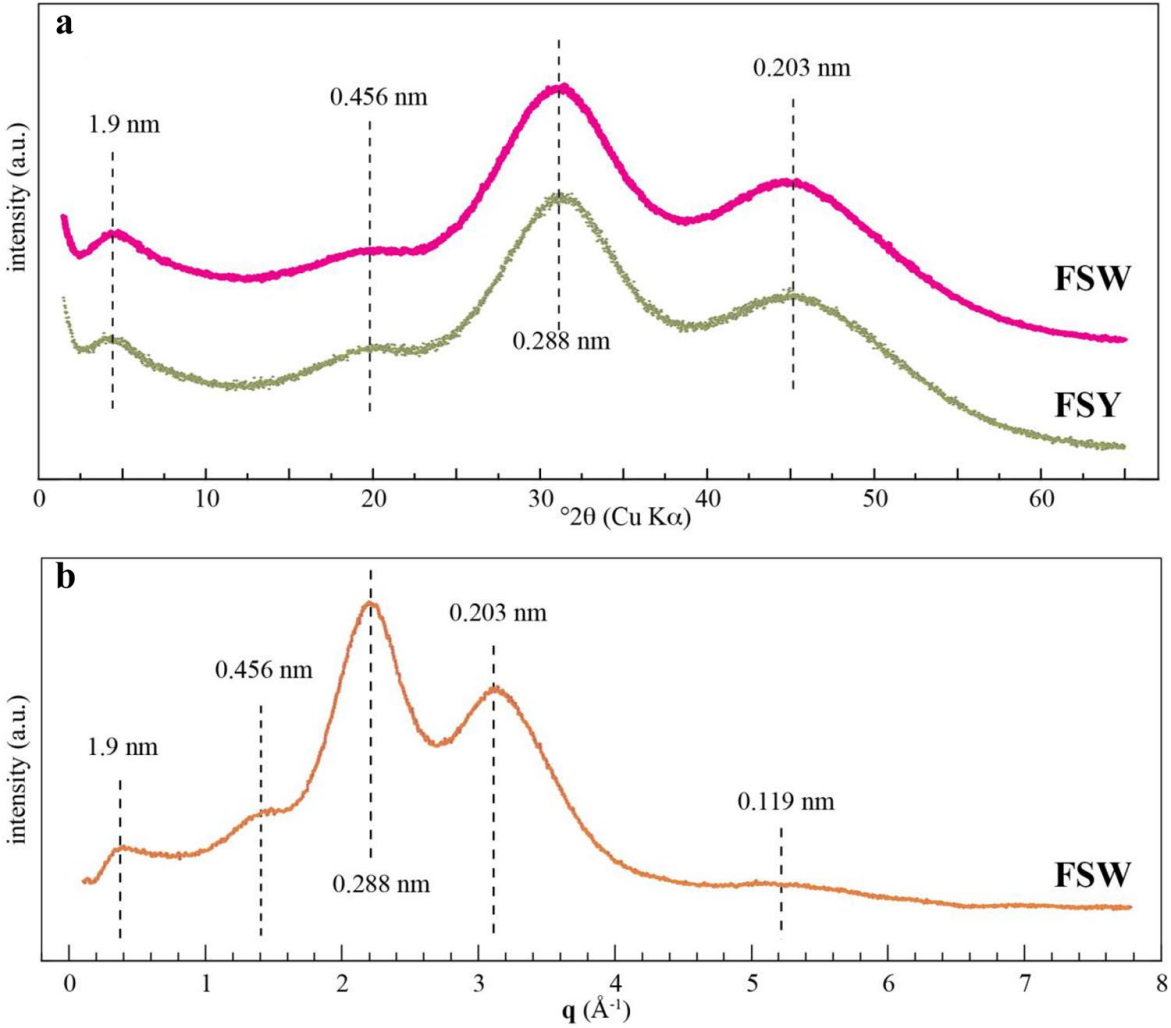
Powder XRD patterns from representative HACC samples from the Arizona (FSW) and UK (FSY) *Fuligo septica* peridia. The FSY pattern is from the same aethalia shown in Fig. 1b. (**a**) XRD patterns of the as-collected material. (**b**) XRD pattern from FSW after background subtraction and intensities plotted as a function of scattering vector **q (**Å^−1^**)**.

Multiple peridial samples were examined by powder XRD: all patterns lack the sharp intense Bragg reflections characteristic of crystalline phases, and are instead dominated by five broad, but well-defined maxima at ~ 1.9, 0.456, 0.288, 0.203, and 0.119 nm, and weak oscillations at higher d-spacings (Fig. 3). The high signal-to-noise ratio of our powder XRD patterns, compared with published data, was possible because of the large amount of pure ACC produced by the slime mold, and its exceptional stability in air. Except for the ~ 1.9 nm reflection, the diffraction patterns are similar to those for synthetic and biogenic ACC^5,7,27,42–44^. The prominent ~ 1.9 nm has not previously been reported and suggests ordering at the ~ 2 nm scale.

**Figure 4:**
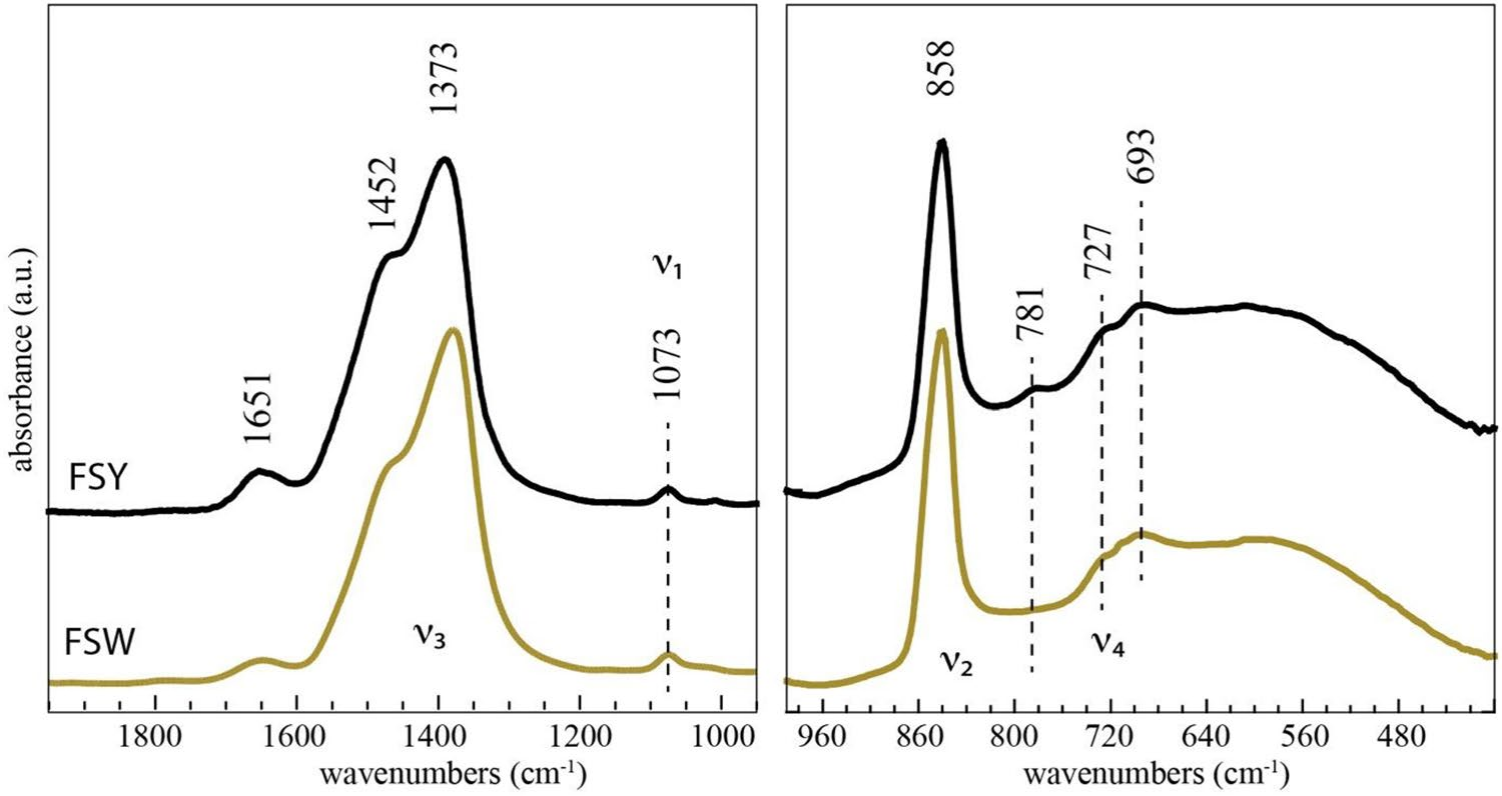
Selected regions of the IR spectra of FSY (sample FS1) and FSW (sample FSW4) HACC acquired at room temperature. Full spectra are shown in the supplementary information (Figs. S4, S16).

The FTIR spectra of the peridia (Figs. 4, S4, S5) show the characteristic bands of ACC, with spectra similar in shape to biogenic (e.g., Fig. 2a, b in Addadi et al.^24^) and synthetic material (Radha et al.^7^ supplement). Most notable is the split of the asymmetric stretch of the carbonate ion at 1373 and 1452 cm^−1^ (ν_3_), and the carbonate out-of-plane bending absorption at 858 cm^−1^ (ν_2_). The FSY and FSW peridia spectra lack the distinctive ν_4_ band for crystalline CaCO_3_, i.e., 712 cm^−1^ for calcite and 744 cm^−1^ for vaterite. Instead, the spectra display low-intensity bands at 727 and 693 cm^−1^ that sit on a broad band centered near 600 cm^−1^. The IR spectrum shows the broad band between 2750 and 3800 cm^−1^ from absorbed and structural water, on which are superimposed weak absorption bands for organic material (inset Fig. S4). In comparison, aged samples collected in the desert show FTIR spectra with characteristic absorption bands for crystalline CaCO_3_ (Figs. S4, S5).

### ACC stability under ambient conditions

Under typical laboratory conditions, the slime mold ACC is stable. For example, the XRD patterns of the FSW samples acquired within hours of formation compared with those stored in the laboratory for 2 years are identical. The moisture stability of the FSY and FSW was also followed through multiple sessions of wetting and drying. The FSY ACC diffraction pattern was unchanged, even after several 24 h wetting and drying sessions. However, the FSW ACC showed the appearance of calcite reflections after the sample was placed in a container with 100% relative humidity (RH) for 24 h. These reflections became more intense with each 24 h session at 100% RH, though no reflections for vaterite were noted (Fig. S6). In contrast, FSW samples collected after weeks to months of natural desert weathering are dominated by vaterite with minor calcite, to those dominated by calcite with minor vaterite: one peridium is composed of monohydrocalcite with minor amounts of calcite (Fig. S7).

### Thermal response

**Figure 5:**
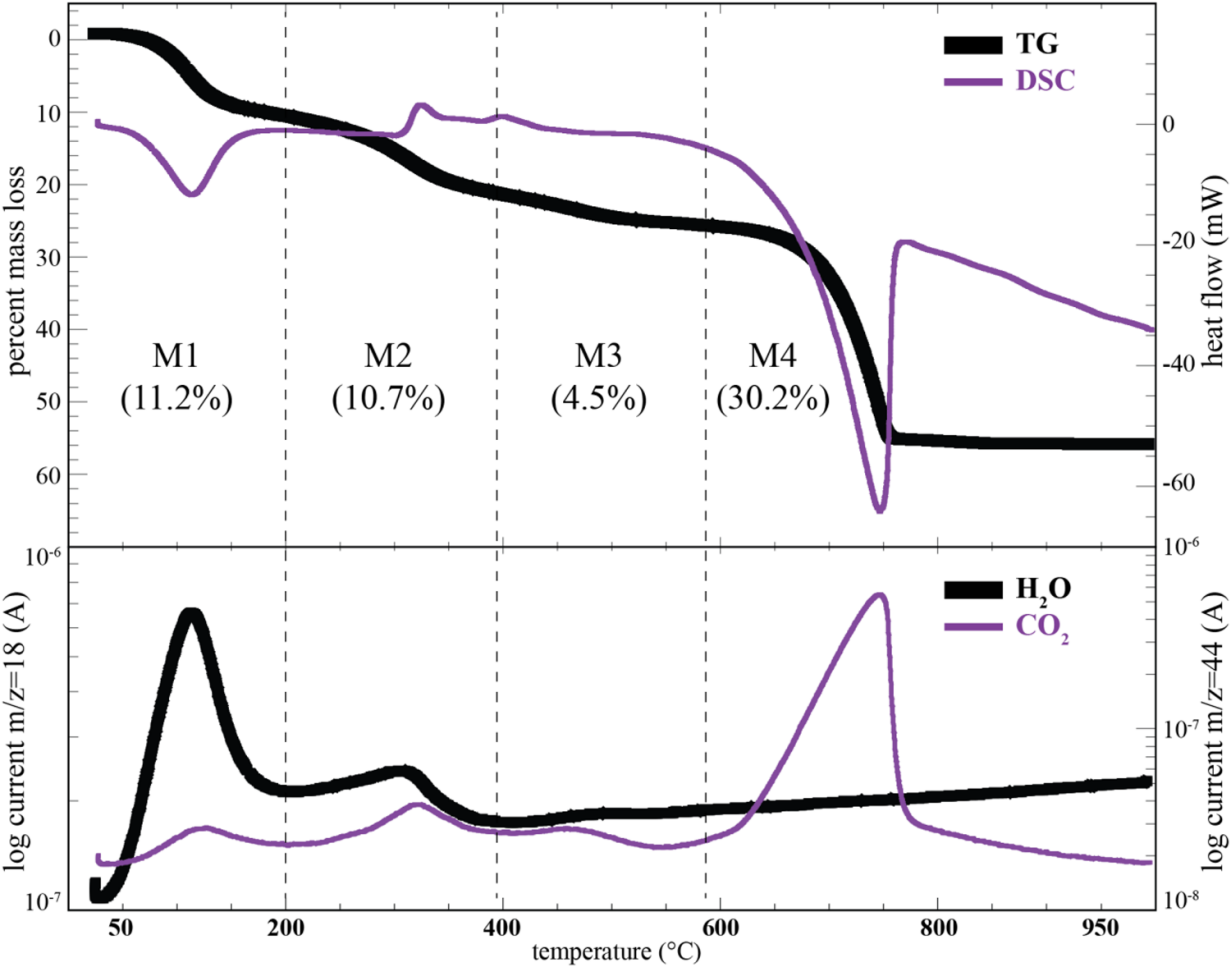
Thermal (TG and DSC) and EGA data for the FSY HACC. M1 to M4 refer to the four distinct mass-loss ranges discussed in the text, and the corresponding percent mass loss within each range in parentheses. The EGA data (bottom panel) show the ion curves for m/z = 18 (H_2_O^+^) and m/z = 44 (^12^CO_2_^+^) on a log intensity scale.

The TG curves for the slime mold ACC heated under a He atmosphere show four distinct mass-loss steps, M1 to M4 (Figs. 5, S8, S9). The total mass losses for the FSW and FSY samples heated to 1000 °C were typically between 52 and 57%. The DSC curves show an endothermic peak at ~ 100 °C, corresponding to dehydration of the ACC and loss of loosely bound water (Fig. 5): we refer to the ACC prior to the loss of this water as hydrated ACC (HACC). The TG loss below 200 °C is between 10.5 and 10.9% for the FSW samples (samples FSW21 and FSW18) and 11.2% for FSY. The EGA ion curves show that the mass loss below 200 °C is dominated by the evolution of H_2_O with minor CO_2_ (Figs. 5, S10, S11). In region M2, the mass loss is dominated by H_2_O (3.8%), CO_2_ (Fig. S11), and organic molecules (Fig. S10d): the intense m/z = 30 signal matches that of formaldehyde (see Table S7 for additional ion peak assignments). During the M3 mass-loss step (between 396 and 584 °C), organic matter is pyrolytically degraded (Fig. S10), with the evolution of a range of organic-bearing species, with minor H_2_O and CO_2_ (Fig. S10). Above 584 °C, calcium carbonate decomposes, and the ion signals are dominated by m/z = 44 corresponding to CO_2_ (Figs. 5, S10). The FSY HACC also changes color during the heating and decomposition process and is black at 500 °C (Fig. S12). This color change may reflect the decomposition of the organic matter to sooty black carbon.

### Quantity of water and organic matter

The FSY and FSW HACC show TG mass losses up to 200 °C of ~ 11 wt%, the majority of which is H_2_O. For example, of the 11.2% mass loss for FSY below 200 °C (Fig. S11), 10.9 ± 0.1% is from H_2_O, and the rest is CO_2_ (see Supplementary section—Water Calculation from TG-DSC-MSEGA measurements). A further 3.8% H_2_O is released in region M2 (Fig. S11), though only 3.0% up to the crystallization temperature onset at 322 °C (see discussion below). However, considering the composition of the FSY HACC as inorganic Ca–C–O–O–O–H–H–O (equivalent to monohydrocalcite: CaCO_3_·H_2_O) + remaining C–H–N–O, then 13.72 wt% water loss (corresponding to 1 mol of water) is expected (Tables S3, S4). The lower-than-expected amount of water released below 200 °C for FSY, i.e., 1CaCO_3_:nH_2_O ratio with n < 1, may indicate significant molecular H_2_O retained above 200 °C, but released before and during crystallization of the ACC starting near 322 °C. Such a scenario is borne out by the TG data, which shows a total H_2_O release up to 322 °C of 13.9%. While the low-temperature water-loss peak has a maximum just above 100 °C, which then drops precipitously with a plateau near 200 °C, H_2_O continues to be released with increasing temperature and peaks near 320 °C, with little H_2_O detected above 400 °C (Figs. S10a, S11). These data suggest that the water released between 200 and 320 °C has several sources including molecular H_2_O and that bound to the organic compounds.

The FTIR spectra show weak absorption bands between 2800 and 3000 cm^−1^ attributed to organic matter associated with the HACC (Fig. S4). The breakdown of this organic matter is also detected by EGA during heating of the HACC (Fig. S10). Samples heated in air have a higher mass loss than those heated under an inert atmosphere (Fig. S13). For example, FSY shows higher mass losses when heated in air between 200 and 575 °C. In this temperature range, 19% of the mass is lost in the case of samples measured in air, and 15.1% when heated under He. This ~ 4% difference sets a minimum in the FSY sample on the organic content as some is pyrolytically degraded under He. The quantity of organic matter can also be estimated from the compositional data (Tables 1, S3 to S6). Assuming an inorganic formula for FSY as (Ca,Mn)_1_CO_3_·H_2_O, then the remainder is assumed to be organic and has the composition C_0.60_O_0.09_H_1.41_N_0.14_, which is 9.13% of the original HACC mass (Tables S3, S4). Similarly, the composition for FSW21 (Table 1) suggests it contains 9.83% organic matter (Tables S5, S6). Thus, the TG data is consistent with the *F. septica* HACC containing between approximately 4 and 10% organic matter.

Despite the elemental and bulk compositional similarities between the *F. septica* HACC and monohydrocalcite (Table 1), their thermal behaviors differ significantly. Monohydrocalcite typically dehydrates by 226 °C, with mass loss of 15.25% H_2_O, corresponding to CaCO_3_·H_2_O → CaCO_3_ + H_2_O, and 37.26 wt% mass loss above 530 °C for CaCO_3_ → CaO + CO_2_^45,46^. Monohydrocalcite shows minimal mass loss below ~ 180 °C, and between ~ 200 and 500 °C. In contrast, the slime mold HACC shows significant mass loss below 180 °C, and also between 200 and 500 °C. The slime mold thermal data are consistent with a range of H environments, similar to that detected in ACC by Nuclear Magnetic Resonance spectroscopy^16^, viz., fluid-like H_2_O, rigid structural H_2_O, restrictedly mobile H_2_O, and hydroxyl. Our mass loss up to 200 °C is dominated by the loss of fluid-like H_2_O.

### Laboratory crystallization of the HACC

The powder XRD data show onset of crystallization of the DACC between 300 and 350 °C (Fig. S14). Similarly, SAED patterns and FTIR spectra of the HACC heated at 208 °C show an amorphous material, but at 362 °C the patterns and spectra show partial crystallization, and at 500 °C the spheres are calcite (Fig. S15 to S17). At 362 °C, the BFTEM images show that the newly formed calcite is ~ 40 nm, which is similar in size to the electron-dense aggregates imaged by BFTEM in the nanospheres (Fig. 2c, S3). By 500 °C, the 40-nm nano-objects have organized into micron-sized grains of calcite (Fig. S15). The spherical morphology is preserved during the transition (Fig. S15). Individual spheres show sharp extinction when viewed with crossed-polarized transmitted light, indicating that each is a single crystal that preserves the original spherical morphology.

**Figure 6:**
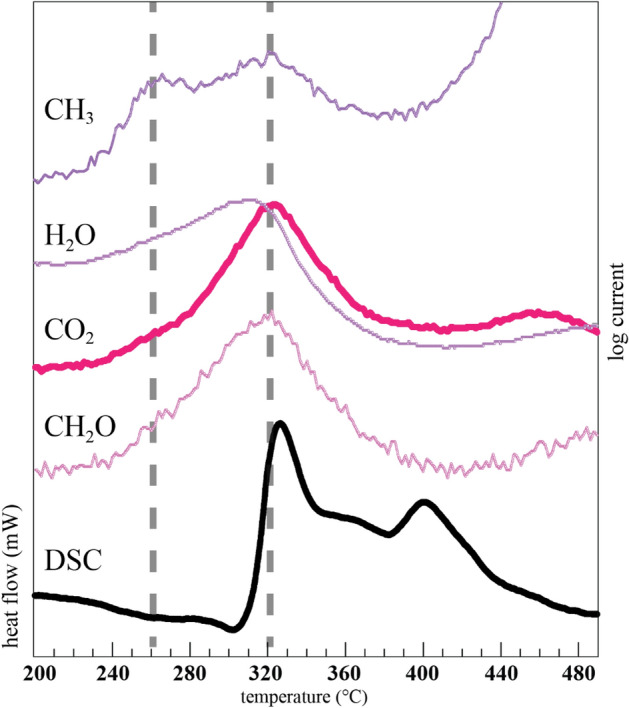
Comparison of the exothermic DSC region (in mW) for the FSY sample run under He (bottom curve) compared with selected EGA profiles (log current) corresponding to CH_2_O^+^ (m/z = 30), ^12^CO_2_^+^ (m/z = 44), H_2_O^+^ (m/z = 18), and CH_3_^+^ (m/z = 15). Additional potential gas assignments are listed in Table S7. All profiles are scaled and shifted along the y-axis so as to show the correspondence between the peak maxima. The absolute EGA scales are shown in Fig. S10.

The DSC profiles for HACC samples examined under flowing He atmosphere show peaks for exothermic reactions between 300 and 480 °C (Fig. 5). The FSY sample shows prominent maxima at 327 and 401 °C in the raw thermogram. Most of the FSW samples similarly reveal two well-resolved exothermic maxima near 338 and 414 °C (Fig. S18), though one sample (FSW21) lacks the 338 °C maximum. The EGA data show a maximum in the evolution of several gases just prior to the first exotherm maximum for both the FSY (Fig. 6) and FSW (Fig. S19) HACC. Many of the gases associated with organic molecules show a two-peaked evolution with maxima at ~ 260 and ~ 320 °C, whereas water and CO_2_ show a more gradual increase in signal intensity starting around 240 °C, with a maximum near 320 °C. The first DSC exotherm between 327 and 338 °C is within the crystallization range identified by powder XRD, and attributed to the crystallization of dehydrated ACC^7,17,42,47^, although this maximum can occur over a range of temperatures^16^. Some biogenic ACC shows two exothermic peaks in the 330–370 °C range^48^. For example, the “Gastrolith ACC 2” DSC curve from^48^ shows two exothermic peaks, of which one may be attributed to the breakdown of chitin^49^. Between the first and second exotherm, the slime mold ACC continues to lose mass, typically between 1.3 and 3.5 wt%. However, additive free ACC does not typically show mass loss following the exothermic event near 340 °C, consistent with a solid-state transformation to calcite as no remaining water is available^7,17^.

Our data suggest the breakdown of an organic framework to the DACC starting near 240 °C, culminating with a maximum in several EGA signals just prior to the first DSC exotherm, with partial crystallization. The powder XRD data for the sample at 350 °C still show a significant amorphous contribution (Fig. S14). By 365 °C, the powder XRD pattern shows intense reflections for calcite that sit on a less intense amorphous background. Optical microscopy shows that at 365 °C, approximately 20% of the spheres are still isotropic. The DSC maximum near 400 °C likely corresponds to the onset of crystallization of the remainder of the still amorphous part of the ACC, as optical microscopy and powder XRD of samples heated at 446 °C show calcite only.

### Multi-scale structure of the slime mold HACC

Our data for the *F. septica* HACC is consistent with the following structural spatial scales, viz., (1) the 500- to 1500-nm-sized sphere; (2) tens-of-nanometer-sized clumping within the spheres; (3) an ~ 2-nm-scale ordering as revealed by the powder XRD; and (4) the short-range ordering that gives rise to the bulk powder XRD pattern. Some of the structural characteristics of the HACC may be explained by the formation mechanisms of the individual spheres. Transmission electron microscopy shows the aggregation and excretion of calcium during sporogenesis in the slime mold *Physarella oblonga*^28^, which is in the same family as *F. septica*, the Physaraceae^50^. There is a massive elimination of the protoplasmic Ca as the slime mold transforms from the mobile plasmodium to the sessile fruiting body. During sporogenesis, electron microscopy reveals the formation and aggregation of tens-of-nanometers-sized Ca-rich, electron-dense, membrane-bound intracellular grains. These grains aggregate and assume their final spherical shape as they grow and are expelled and form the peridium as a fully formed 500- to 1500-nm-sized sphere^28^. This membrane-bound aggregate model is consistent with our BFTEM of the *F. septica* spheres, which internally show tens-of-nanometer-scale electron-dense clumping (Figs. 2c, S3), suggesting that they also aggregated from tens-of-nanometers-sized Ca-rich, electron-dense, membrane-bound intracellular grains. However, the grains themselves are amorphous as the powder XRD profiles do not show reflections indicative of crystalline ordering at the tens-of-nanometer scale.

The origin of the 1.9 nm powder XRD maximum is less well understood. This maximum becomes indistinct, or is absent, for samples heated to ~ 100 °C, or stored over the aggressive drying agent P_2_O_5_. Thus, the 1.9 nm maximum may reflect ordering at the ~ 2-nm scale that is absent after loss of the weakly bound water. Ordered mesoporous material can give rise to low-angle maxima that reflect the pore-to-pore distance as well as the pore diameter^51^. Thus, the ~ 1.9 nm maximum suggests an ordered mesoporous structure to the *F. septica* HACC that is readily lost on removal of the loosely bound water.

**Figure 7:**
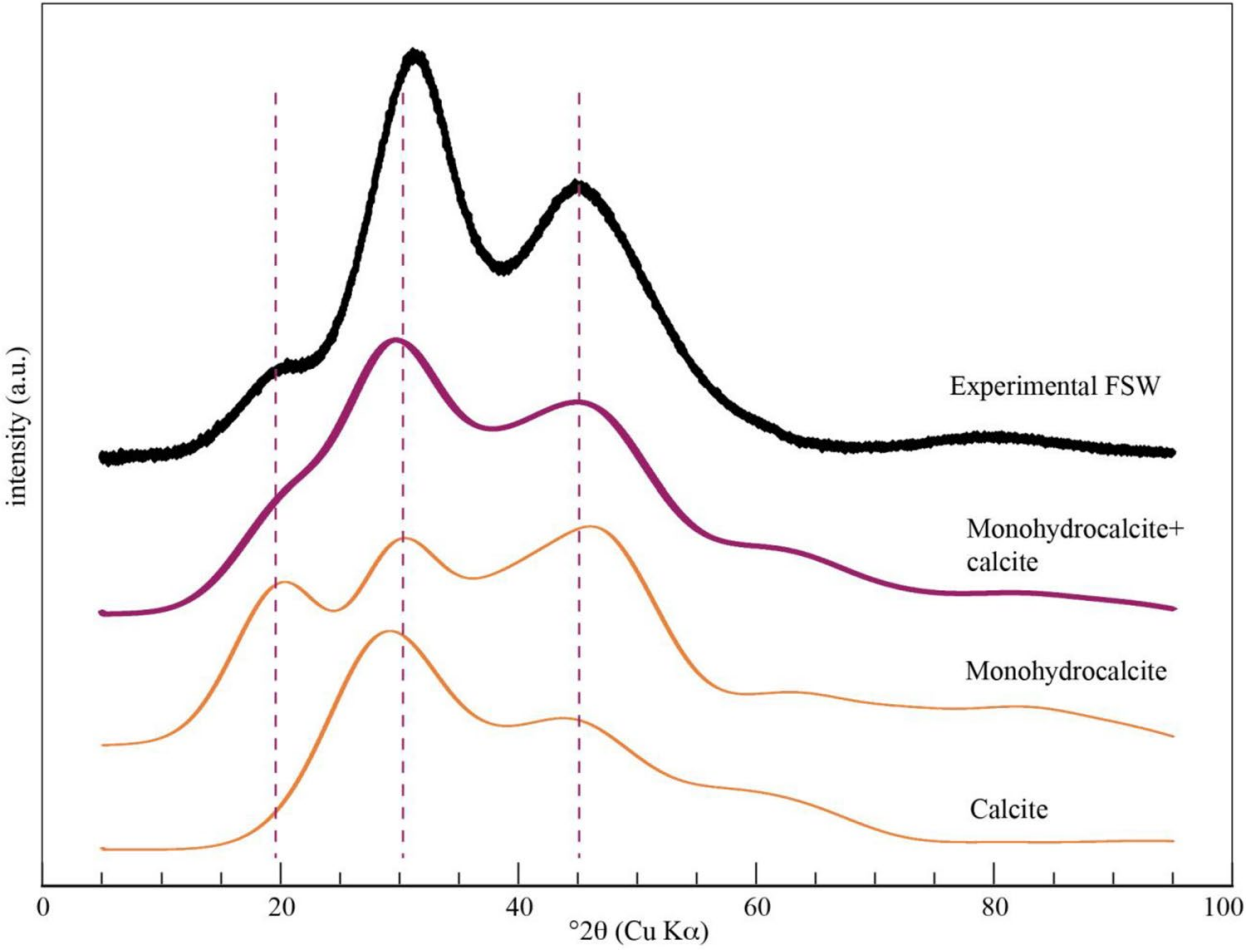
Comparison of the experimental XRD diffraction profile for the FSW HACC (top pattern) compared with simulated patterns for 0.8-nm calcite, 1-nm monohydrocalcite, and a linear sum of the two (Monohydrocalcite + calcite). The measured background below the experimental pattern has been subtracted as well as the contribution from the 1.9 nm peak.

The powder XRD patterns for the room temperature and heated *F. septica* HACC lack discrete reflections for crystalline phases, and instead present broad maxima that reflect the short-range order within the spheres. These maxima represent the average interatomic distance scattering within the material and are indicative of short-range order. Rez et al.^8^ show a strong match between the SAED patterns from synthetic and biogenic ACC and calculated patterns for random packing of ~ 1-nm-sized calcite crystals. Similarly, we compare the powder XRD patterns for the *F. septica* HACC with the simulated scattering profiles for ~ 1-nm-sized particles of anhydrous and hydrated CaCO_3_ polymorphs (Fig. 7, S20). Only the simulated pattern for monohydrocalcite shows four oscillations that match the 0.456, 0.288, 0.203, and 0.119 nm maxima from the *F. septica* HACC powder XRD patterns. The simulated pattern for monohydrocalcite further shows a weak oscillation near 0.1467 nm which, if present in the experimental pattern, is obscured by the tail of the intense 0.288 nm maximum. However, the 0.288 nm maximum in the experimental pattern is considerably more intense than the corresponding maximum in the simulated pattern for monohydrocalcite. The intensity of this maximum can be simulated by assuming an HACC structure with both monohydrocalcite- and calcite-like nano-structural ordering (Fig. 7). The main maximum for the HACC at 0.288 nm is at a higher d-spacing than that predicted by the simulations. A similar situation was shown between the calculated patterns for calcite and biogenic calcite, which Rez et al.^8^ attributed to the contraction of the nanocrystals relative to the bulk calcite. The simulations lend support to a structure composed of ~ 1-nm-sized diffracting domains, as the simulated patterns change dramatically just by doubling the particle size, with sharpening of the maxima and appearance of new peaks that are not present in the experimental patterns (Fig. S21).

### Location of organic matter

Organic material is present in the peridium and hypothallus of *F. septica*^29,35^. Nelson and Orlowski^35^ show a predominance of protein with significant contributions of galactose and galactosamine polymer. This protein is dominated by a single M_r_ 14,000 polypeptide. Much of this organic matter likely arises from the slime produced by multinucleate slime molds^52^. Slime, which is an exopolysaccharide, isolated from two multinucleate slime molds consist largely of carbohydrates, proteins, and various sulfate groups, with galactose, glucose, and rhamnose the dominant monomers of the carbohydrates^52^.

Our TG and elemental data are consistent with the bulk HACC of *F. septica* containing up to 10 wt% organic matter. This quantity is similar to the 10.6 wt% organic matter determined for *F. septica* by Chapman et al.^29^, which is divided between protein (6.8 wt%) and carbohydrates (3.8 wt%). The internal clumping observed by BFTEM in the spheres (Figs. 2c, S3) suggests the presence of less electron-dense material between the clumps: this material is likely the location of organic material deposited on the clumps during their intracellular formation and aggregation (e.g., see Figs. 14 to 18 in Bechtel and Horner^28^). In addition, the TEM images also show low-contrast material between and on the spheres (Fig. S15e) that is likely organic, and most probably dried slime. Our present data cannot ascertain the distribution and location of individual proteins and carbohydrates within and on the HACC spheres. However, it is likely that the abundant proteins and/or carbohydrates in the *F. septica* peridia^29^ are responsible, at least in part, for the stability of the HACC.

## Conclusions

The powder XRD, electron diffraction, and IR data, together with the lack of optical birefringence are consistent with the *F. septica* HACC spheres as an amorphous material with sub-nanometer-sized diffracting domains. The XRD simulations are consistent with an HACC that possesses short-range order, with both monohydrocalcite- and calcite-like nano-structural properties, possibly reflecting a range of coordination environments from six to eight O around Ca. The EGA data are consistent with the breakdown of an organic framework to the HACC starting near 240 °C, culminating with a maximum in several EGA signals just prior to the first DSC exotherm which is associated with partial crystallization. Together, the powder XRD, EGA, and elemental data suggest a structure dominated by ~ 1-nm-sized monohydrocalcite- and calcite-like units separated by organic material. Further, the ~ 1.9-nm-sized spacings present in the HACC XRD patterns suggest ordering at this scale that is disrupted as the mobile water is released. Previous work on ACC suggests that crystallization occurs from an intermediate anhydrous ACC. However, the slime mold DACC is not strictly anhydrous as ~ 3 wt% water is released after the loss of the loosely bound water and before the first crystallization exotherm. This retained water is supported by the IR spectra that still show strong, broad H_2_O absorption bands after heating to 208 °C.

The transformation and crystallization of the *F. septica* HACC by laboratory heating differs significantly from that observed in naturally weathered samples collected in the desert. Many examples of *F. septica* collected within weeks to months after their formation in the desert were vaterite-rich, with one dominated by monohydrocalcite. These mineralogical differences suggest that the crystallization mechanism(s) via laboratory heating versus natural weathering are not the same. In the desert environment, the peridial HACC, which sits on the ground, will experience significant diurnal temperature and humidity variations^53^: these variations could drive the reorganization of the HACC into different crystalline anhydrous or hydrous calcium carbonates, possibly as the stabilizing organic molecules are leached from the peridium. These data suggest that the transformation pathway is not necessarily pre-determined by the initial HACC structure, but instead by environmental or laboratory conditions, a conclusion consistent with previous findings^17^.

A major finding of our study is that the HACC precipitated by the slime mold *F. septica* is indefinitely stable under normal laboratory conditions. We propose that the intimate association of the organic matter with ~ 1-nm-sized monohydrocalcite- and calcite-like units inhibits its crystallization. The composition, structure, and thermal behavior of the HACC precipitated by *F. septica* collected over 8000 km apart, and in markedly different environments, suggests a common structure, as well as similar biochemical and biomineralization formation mechanisms.

## Experimental section

### Field observations

*Fuligo septica* is a cosmopolitan species^38^. Specimens collected in southern Arizona are commonly found after summer rains. The author observed specimens from the arid parts of southwestern Arizona to the more mesic mountain regions in the eastern part of the state. It is locally abundant, for example 103 aethalia were counted in a 15 × 5 m area (33° 16′ 33.14″ N, 111° 9′ 57.25″ W, August 2018) under mesquite trees. Several hundred aethalia were found amongst perennial bushes and trees along a 1 km stretch of a dry wash (33° 1′ 58.15″ N, 112°14′ 35.99″ W, July 2017) southwest of Phoenix. Aethalia are also common after summer rains in the senior authors garden and on the grounds of Arizona State University. The plasmodium emerges from the damp ground during the relative cool of the night and moves to an exposed position where it forms an aethalium. By morning, the aethalium is fully formed. The aethalia are typically pulvinate, 3 to 15 cm in diameter, and up to 2 cm thick. A cross section of the aethalium shows a dome-shaped peridium of white, soft, chalky material coating the spore mass (Fig. S1). The peridium and spore mass sit on the hypothallus. The peridium from eight separate aethalia of *F. septica* were collected during June 2018 in the 28 hectare Highgate Wood of north London (51° 35′ 0.38″ N, 0° 8′ 59.42″ W). The aethalia are pulvinate, bright canary yellow, with the largest to 7 cm. The peridium is up to 3 mm thick and separated easily from the spore mass. The aethalia were all whole and undisturbed and it is estimated that they were collected within a few days of having formed. The Arizona and UK specimens studied here match the description for *F. septica* in^38^.

### Separation of the ACC

The peridium from the Arizona (FSW) aethalia was easiest to collect from the still-moist samples soon after collecting in the early morning. The white but damp peridium has a consistency of whipped cream cheese, which is easy to collect with a small spatula from the edge of aethalia. In this way, up to 200 mg of pure, dried ACC could be separated from a single aethalia. The peridium can also be collected from the dry aethalia, although care is needed so as to not incorporate the underlying spores in the sample. In contrast, the peridium from the UK (FSY) samples separated easily from the spore mass as porous yellow chunks up to 1 cm across (Fig. S2).

### Proton-induced x-ray emission

Nondestructive elemental analysis of slime mold ACC was undertaken by proton-induced x-ray emission (PIXE) spectroscopy. Proton beams were accelerated at low energy (1.90 MeV), with a 1.7 MeV Tandetron tandem accelerator (Cockroft-Walton type manufactured by General Electric). The proton beam of 1 × 1 mm crosses a 7.8-micron-thick kapton foil window before entering the sample chamber and striking the sample. The sample chamber is evacuated to low vacuum to avoid air signal and x-ray absorption. A Canberra Si(Li) detector (detector resolution at the 5.9 keV line is 168.0 eV) is placed at 47° from the normal of the sample surface, which is oriented at 45° with respect to the incoming proton beam. No filters were used in front of the detector for the low-energy, light-element analyses. The proton current incident on the sample was adjusted to ~ 0.5 nA.

PIXE was used to measure element concentrations of atomic number 11 (sodium) and greater. The spectra were acquired from areas ~ 1 × 1 mm on pressed 2-mm-diameter discs of ACC. Each spectrum was acquired for a total of 10,000 counts. The PIXE data were processed with the GUPIX software (www.physics.uoguelph.ca/PIXE, updated 2005). For standardization the instrumental constant *H* (solid angle and correction factor) was determined using the GUPIX database (cross-sections, fluorescence and Coster–Kronig probabilities, stopping powers and attenuation coefficients) for the range of element in the NIST biological reference material Bovine Liver (SRM-1577) and whewellite. The bovine standard was run, and *H* determined prior to each ACC analysis. The C-H-N elemental data for the ACC (see below) were used to define the matrix for the GUPIX calculations.

### CHN analysis

Bulk carbon, hydrogen, and nitrogen were determined using a Perking Elmer 2400 Elemental Analyzer in the Metals, Environmental and Terrestrial Analytical Laboratory at Arizona State University. Approximately 5 mg of powder was used for each analysis. The samples were loaded into tin cups and flash heated to 1760 °C. The resulting gases were chemically scrubbed of the halogens and S and separated in a GC column. Detection is conducted by a thermal conductivity detector.

### Scanning and transmission electron microscopy

Dry precipitates of the *Fuligo septica* HACC were gold coated and imaged with a ZEISS EVO 40 scanning electron microscope operated at 5 keV. TEM data were acquired with a 200 keV Talos Thermo Scientific transmission electron microscope. Grains of the pristine material and samples heated at 208, 361, and 500 °C in an inert atmosphere for ½ h were crushed under ethanol and deposited onto copper grids covered by lacey carbon. We obtained BFTEM, and HAADF-STEM images as well as SAED patterns. The elemental composition of the grains was measured with a “Super-X” detector system having four silicon drift detectors built into the microscope column.

### Powder X-ray diffraction and simulations

Powder XRD patterns were acquired with a Rigaku MiniFlex 600 diffractometer. This diffractometer is operated with Cu *Ka* radiation and is equipped with a post-diffraction graphite monochromator and automatic divergence slit system. Data was typically acquired in step scan mode at 0.02° steps, and 30 to 60 s/step. Samples, typically weighing ~ 10 mg, were mixed with a small drop of methanol forming a slurry. The resulting slurry was pipetted and spread into a thin, smooth film on a low-background, single-crystal, quartz plate. This slurry was dried rapidly (~ 5 s) under blowing warm air forming a thin film. In order to demonstrate that the methanol treatment did not affect the shape of the XRD profile, a sample was run as a dry powder sprinkled onto the quartz plate. The XRD pattern from the dry powder pattern and the thin film formed from the slurry were identical, showing that the methanol does not affect the slime mold HACC structure.

Samples, weighing ~ 40 mg each, were heated at 150, 300, 350, and 500 °C in air for ½ h, and two samples of ~ 20 mg were heated at 365 and 446 °C under flowing He for ½ h. The specific heating temperatures were guided by the dominant changes observed in the TG-DSC data. The heated samples were deposited onto the quartz plate and the XRD pattern initially acquired rapidly (1/2 h) and then over an extended time period (20 h) to ensure that initially heated material remained stable. Samples were also periodically rerun over the course of months to 2 years to check for changes in the overall shape of the diffraction patterns.

The moisture stability of the FSY and FSW samples was investigated by placing the XRD slide with the sample used for powder XRD into a sealed container over water at 50 °C for 24 h. The sample became damp over the 100% RH and a new powder XRD pattern was acquired.

Simulated patterns were calculated with the CrystalDiffract software by CrystalMaker Software Ltd. Patterns were calculated assuming Gaussian profiles, particle sizes were varied as shown in the figures and Iso strain was set to 0%. Ideal lattice parameters were used for the simulations and site occupancies were set at 100%.

### FTIR spectroscopy

FTIR measurements were recorded on a Jasco FT/IR-4600 (Japan) system, equipped with a Jasco ATR Pro One single reflection diamond ATR (attenuated total reflection) accessory (incident angle 45°), and a mid-range MCT (Mercury-Cadmium-Telluride) detector. A spectral resolution of 4 cm^−1^ and co-addition of 128 individual spectra were applied. Prior to the evaluation, an ATR correction (Jasco Spectra Manager version 2, Spectra analysis module version 2.15.11) was performed on the raw spectra.

### TG–DTA/DSC

Thermal measurements were performed on a Setaram LabsysEvo (Lyon, France) TG–DTA/DSC system, in flowing (60 mL/min) purging gas atmosphere [99.9999% purity He /DTA/, 99.999% purity Ar /DSC/ and 99.999% purity synthetic air (20% O_2_ in N_2_) /DSC/ atmospheres]. The sample was weighed into a 100 μL Al_2_O_3_ crucible (the reference crucible was empty) and heated from 25 to 1000 °C with a heating rate of 10 °C/min. Two measurements were done in one type of gas, one with a smaller amount of mass (~ 10 mg) and another one with a larger sample mass (~ 35 mg) in order to enhance the effects on the heat flow signal. The obtained data was baseline corrected and further processed with the thermoanalyzer’s processing software (Calisto Processing, ver. 2.092). The thermal analyzer (both the temperature scale and calorimetric sensitivity) was calibrated by a multipoint calibration method, in which seven different certified reference materials (CRM’s) were used to cover the thermal analyzer’s entire operating temperature range.

### TG-DSC-MSEGA

Thermal measurements were performed on a Setaram LabsysEvo (Lyon, France) TG-DSC system, in flowing (90 mL/min) helium gas (99.9999% purity) atmosphere. The sample was weighed directly into a 100 μL Al_2_O_3_ crucible (the reference cell was empty) and was heated from 25 to 1000 °C with a heating rate of 20 °C/min. The obtained data was baseline corrected and further processed with the thermoanalyzer’s processing software (Calisto Processing, ver. 2.092). The thermal analyzer (both the temperature scale and calorimetric sensitivity) was calibrated by a multipoint calibration method, in which seven different certified reference materials (CRM’s) were used to cover the thermal analyzer’s entire operating temperature range. In parallel with the thermal measurements, the analysis of evolved gases/volatiles was performed on a Pfeiffer Vacuum Omni Star™ mass spectrometric evolved gas analysis system (MS-EGA), which was connected to the above-mentioned thermal analyzer. The gas splitter was thermostated to 230 °C, while the transfer line to the mass spectrometer was thermostated to 220 °C. The temperature of the mass spectrometer gas inlet was programmed to 120 °C. The measurements were done in SEM Bargraph Cycles acquisition mode, where the m/z interval of 11–130 was continuously scanned with a speed of 50 ms/amu. The spectrometer was operated in electron impact mode. The amount of “free” water was calculated by comparing the corresponding areas between room temperature and 200 °C from two standard calibration materials (calcium oxalate monohydrate and potassium bicarbonate) adapted from the work of^54^.

## Supplementary Information

Supplementary Information.
