# Supplementary material for: An exceptionally stable and widespread hydrated amorphous calcium carbonate precipitated by the dog vomit slime mold Fuligo septica (Myxogastria)
Source: Sci Rep. 2022 Mar 7;12:3642. doi: 10.1038/s41598-022-07648-9 (PMC8901774; doi:10.1038/s41598-022-07648-9)
Supplement: Supplementary file 1 — Supplementary Information. [file 41598_2022_7648_MOESM1_ESM.pdf]

An exceptionally stable and widespread hydrated amorphous calcium carbonate precipitated by the dog vomit slime mold *Fuligo septica* (Myxogastria)

Laurence A.J. Garvie[1], Péter Németh[2,3], and László Trif[4]

Supplementary Data and Text

Water Calculation from TG-DSC-MSEGA measurements

The amount of H_2O lost up to 200 °C in FSY was determined from the TG-DSC-MSEGA data based on the adaptation of the method described by Verchovsky et al. (2019), in which several measurements are performed using two high purity reference materials - calcium oxalate monohydrate (AlfaAesar Puratronic, 99.9985% (metals basis)) and potassium bicarbonate (Sigma-Aldrich, ≥99.95% trace metals basis). Water and CO_2 are released from calcium oxalate in two distinct separated mass loss steps, but in one mass loss step in potassium bicarbonate. For the determination of water in the FSY, three different approaches were tried. In the first, TG-DSC-MSEGA measurements were performed with the two standards but with different starting masses. Considering the decomposition reactions for each standard, the amount of H_2O released from the weighted starting reference material was calculated, then the corresponding peaks on the molecular ion's (m/z – 18 for water) ion current curve were integrated. Based on these values, a calibration curve from the mass of H_2O (mg) – peak area (A·s) data pairs was drawn. By determining the area of the peak corresponding to the H_2O loss up to 200 °C in the ion current curve of m/z – 18 for FSY, using the calibration curve's equation (linear fitting, R2 = 0.9899) determined previously, the amount of water lost was calculated. Another approach was to compare the area of all volatiles lost up to 200 °C with the value of the mass lost. For this, the areas (areas of the peaks on the molecular ion's discrete ion chromatograms, m/z – 18 for H_2O and m/z – 44 for CO_2) of all volatiles were determined, which corresponds to the value of mass loss determined in the TGA (11.177%). From this, the contribution of H_2O to the total mass loss was calculated. In the third approach, the area on the ion current curve of a single standard material (where the amount of H_2O released was close to the amount of water released from FSY up to 200 °C) was compared to the area of the H_2O in the FSY sample. By knowing the amount of H_2O formed from the decomposition of the standard, the amount of H_2O was calculated by comparing the two above mentioned areas. The amount of H_2O determined from each approach was: 10.812%, 10.9% and 11.01%, respectively. Averaging these values, the H_2O lost from FSY1 up to 200 °C is 10.9% +/-0.1%.

Verchovsky, A., Anand, M., Barber, S.J., Sheridan, S. & Morgan, G. (2019) A quantitative evolved gas analysis for extra-terrestrial samples. Planetary and Space Science, 181: 104830

Table S1 Element concentrations (in ppm) determined by PIXE analysis for the FSW and FSY HACC. Three separate analyses were acquired for each of the two samples FSW21 and 22 and two for FSY1. Limits of detection in brackets.

Sample	Mg	P	S	Cl	K	Ca	Mn
**FSW21a**	331 (391)	2809 (330)	1982 (168)	465 (168)	940 (331)	318559 (293)	1481 (1066)
**FSW21b**	390 (402)	2576 (309)	1845 (166)	403 (160)	510 (307)	287986 (293)	937 (897)
**FSW21c**	542 (374)	2850 (315)	1846 (158)	415 (161)	710 (318)	306052 (393)	1291 (952)
**FSW22a**	399 (398)	1425 (335)	965 (166)	488 (178)	1118 (338)	331588 (159)	3061 (937)
**FSW22b**	692 (406)	1312 (326)	976 (168)	643 (171)	1310 (312)	318311 (195)	1705 (991)
**FSW22c**	188 (352)	1237 (313)	710 (170)	522 (174)	1093 (322)	320955 (227)	2186 (1059)
**FSY1a**	665 (404)	1976 (282)	837 (173)	1535 (169)	2074 (319)	280817 (245)	33723 (456)
**FSY1b**	540 (385)	1880 (275)	1049 (160)	1582 (151)	1813 (301)	279966 (236)	32902 (923)

Table S2. Elemental concentrations of carbon, hydrogen, and nitrogen (wt%) as determined by CHN analysis from the FSW and FSY HACC.

Sample	Weight (mg)	Carbon	Hydrogen	Nitrogen
FSW21A	4.943	12.49	2.51	1.02
FSW21B	4.874	12.49	2.50	1.00
**FSW21$_{avg}$**		**12.49**	**2.505**	**1.01**
FSW22A	4.983	12.12	2.35	0.88
FSW22B	4.987	12.17	2.35	0.86
**FSW22$_{avg}$**		**12.145**	**2.35**	**0.87**
FSY1A	4.985	14.68	2.62	1.29
FSY1B	5.098	14.57	2.61	1.71
**FS$_{avg}$**		**14.625**	**2.615**	**1.50**

Table S3. Conversion of the FSY composition in wt% (from Table 1) into at% and as total cations equal to one.

Element	wt%	C	m	at%	1Ca+Mn
Ca	28.100	40.078	0.70113	8.9891	0.92043
Mn	3.3300	54.938	0.060614	0.77712	0.079572
O	49.820	16.000	3.1137	39.921	4.0877
C	14.630	12.011	1.2181	15.616	1.5990
H	2.6200	1.0080	2.5992	33.324	3.4122
N	1.5000	14.007	0.10709	1.3730	0.14058

Table S4. Composition of the FSY HACC expressed as Ca-Mn-C-O-O-O-H-H-O (equivalent to $CaCO_3.H_2O$) + remaining C-H-N-O. In this form, the FSY HACC contains 9.13 wt% organic material and 13.72 wt% water.

Element	1(Ca+Mn)	at%	atomic weight	p	wt%
Ca	0.92043	8.9891	40.078	360.27	28.100
Mn	0.079572	0.77712	54.938	42.693	3.3299
C	1.0000	9.7662	12.011	117.30	9.1492
O	1.0000	9.7662	16.000	156.26	12.188
O	1.0000	9.7662	16.000	156.26	12.188
O	1.0000	9.7662	16.000	156.26	12.188
H	1.0000	9.7662	1.0080	9.8443	0.76783
H	1.0000	9.7662	1.0080	9.8443	0.76783
O	1.0000	9.7662	16.000	156.26	12.188
C	0.59900	5.8500	12.011	70.264	5.4804
H	1.4122	13.792	1.0080	13.902	1.0843
N	0.14058	1.3729	14.007	19.231	1.4999
O	0.087700	0.85650	16.000	13.704	1.0689

Table S5. Conversion of the FSW21 composition in wt% (from Table 1) into at% and as total cations equal to one.

A	wt%	Z	m	at%	Ca+Mn=1
Ca	30.500	40.078	0.76102	9.8882	0.99714
Mn	0.12000	54.938	0.0021843	0.028381	0.0028620
O	53.375	16.000	3.3359	43.345	4.3710
C	12.490	12.011	1.0399	13.512	1.3625
H	2.5050	1.0080	2.4851	32.290	3.2562
N	1.0100	14.007	0.072107	0.93691	0.094479

Table S6. Composition of the FSW21 HACC expressed as Ca-Mn-C-O-O-O-H-H-O (equivalent to $CaCO_3.H_2O$) + remaining C-H-N-O. In this form, the FSW21 HACC contains 9.83% organic material and 13.75 wt% water.

Element	Ca+Mn=1	at%	atomic weight	P	wt%
Ca	0.99714	9.8882	40.078	396.30	30.501
Mn	0.0028620	0.028381	54.938	1.5592	0.12000
C	1.0000	9.9166	12.011	119.11	9.1671
O	1.0000	9.9166	16.000	158.67	12.212
O	1.0000	9.9166	16.000	158.67	12.212
O	1.0000	9.9166	16.000	158.67	12.212
H	1.0000	9.9166	1.0080	9.9959	0.76933
H	1.0000	9.9166	1.0080	9.9959	0.76933
O	1.0000	9.9166	16.000	158.67	12.212
C	0.36250	3.5948	12.011	43.177	3.3231
H	1.2562	12.457	1.0080	12.557	0.96644
N	0.094479	0.93691	14.007	13.123	1.0100
O	0.37100	3.6791	16.000	58.865	4.5305

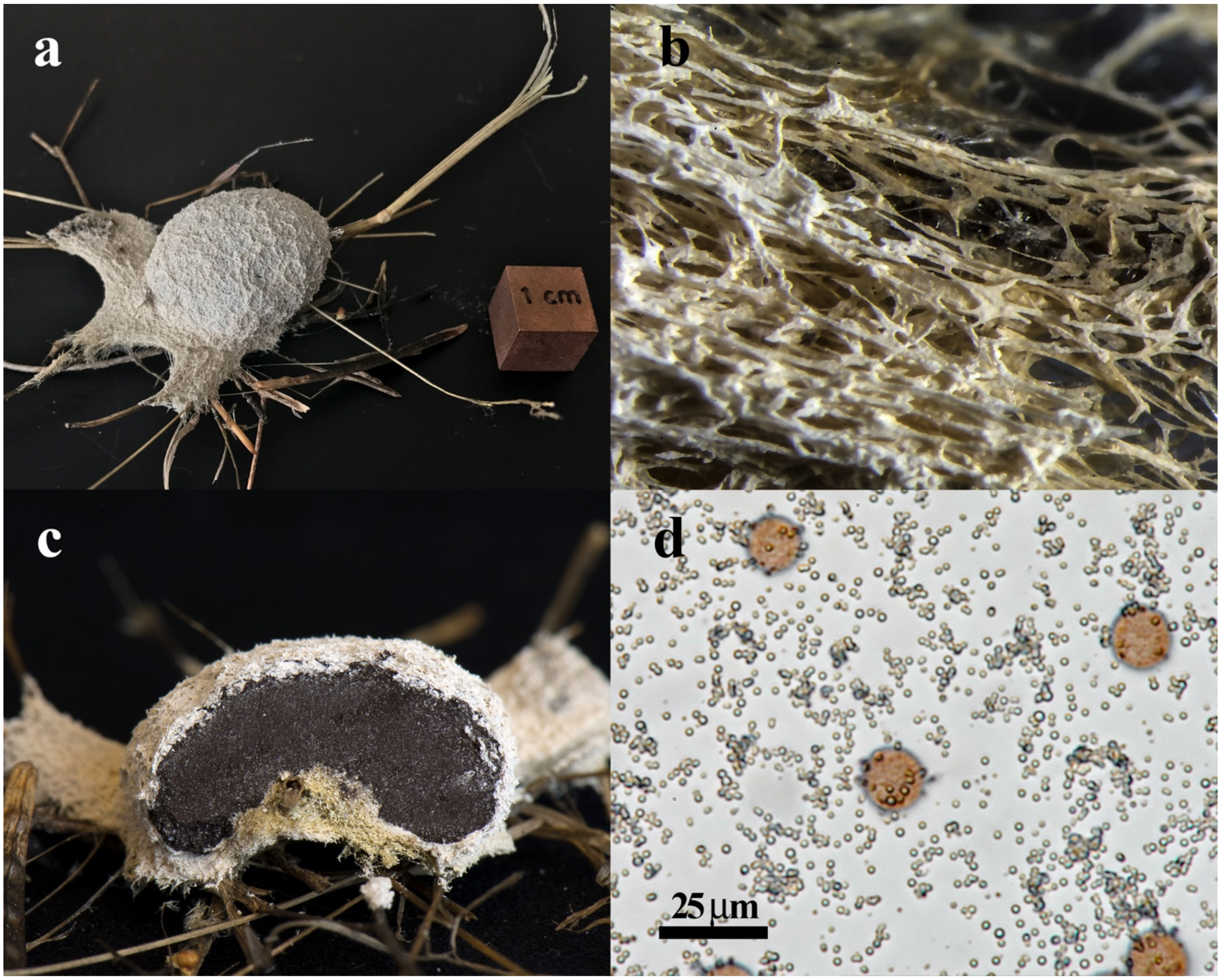

Figure S1. Photographs of a *Fuligo septica* from Arizona (FSW). **a**) Complete aethalium adhering to dried sticks and grass. **b**) Closeup of the peridium. **c**) Cross section of the aethalium showing the dark spore mass and white peridial coating. **d**) Red spores and smaller HACC spheres from the peridium. The spores are ~10 microns in diameter.

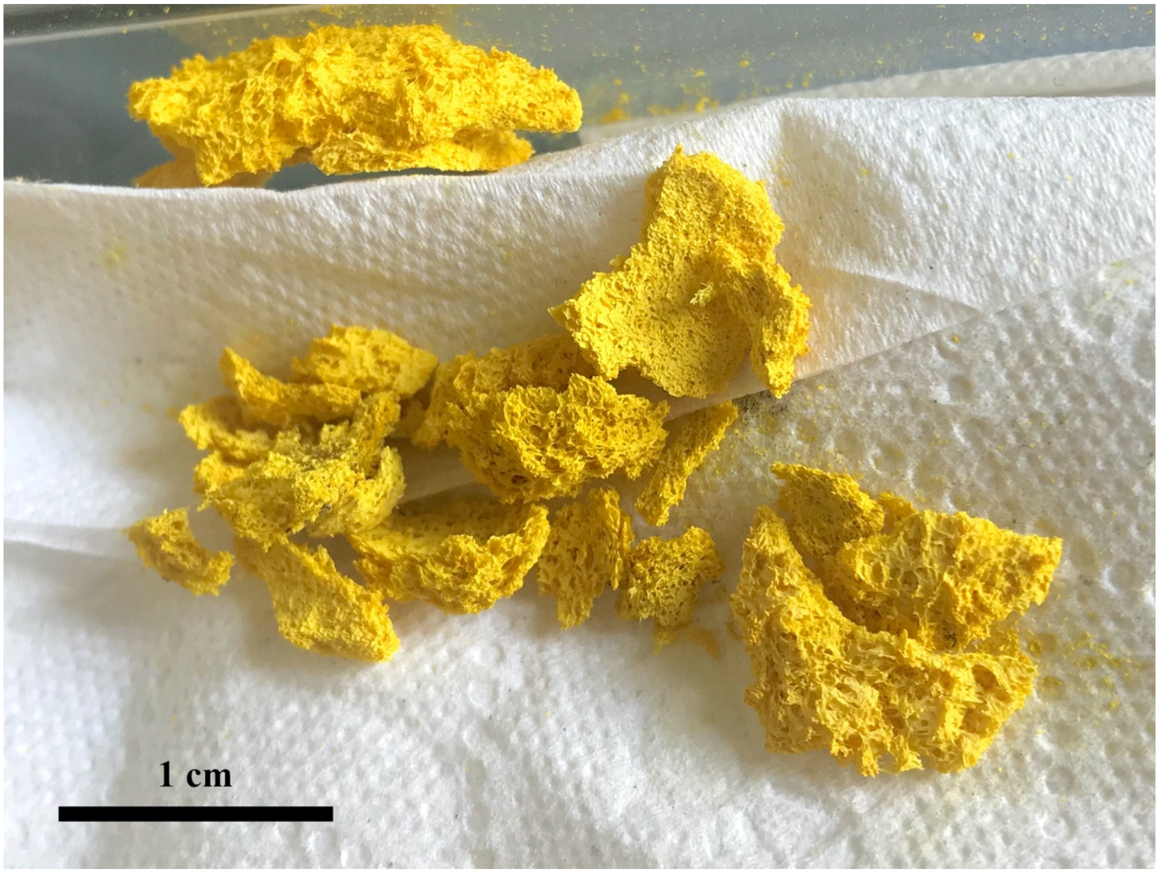

Figure S2. Fragments of the canary yellow peridium from the FSY *F. septica* shown in Fig. 1b.

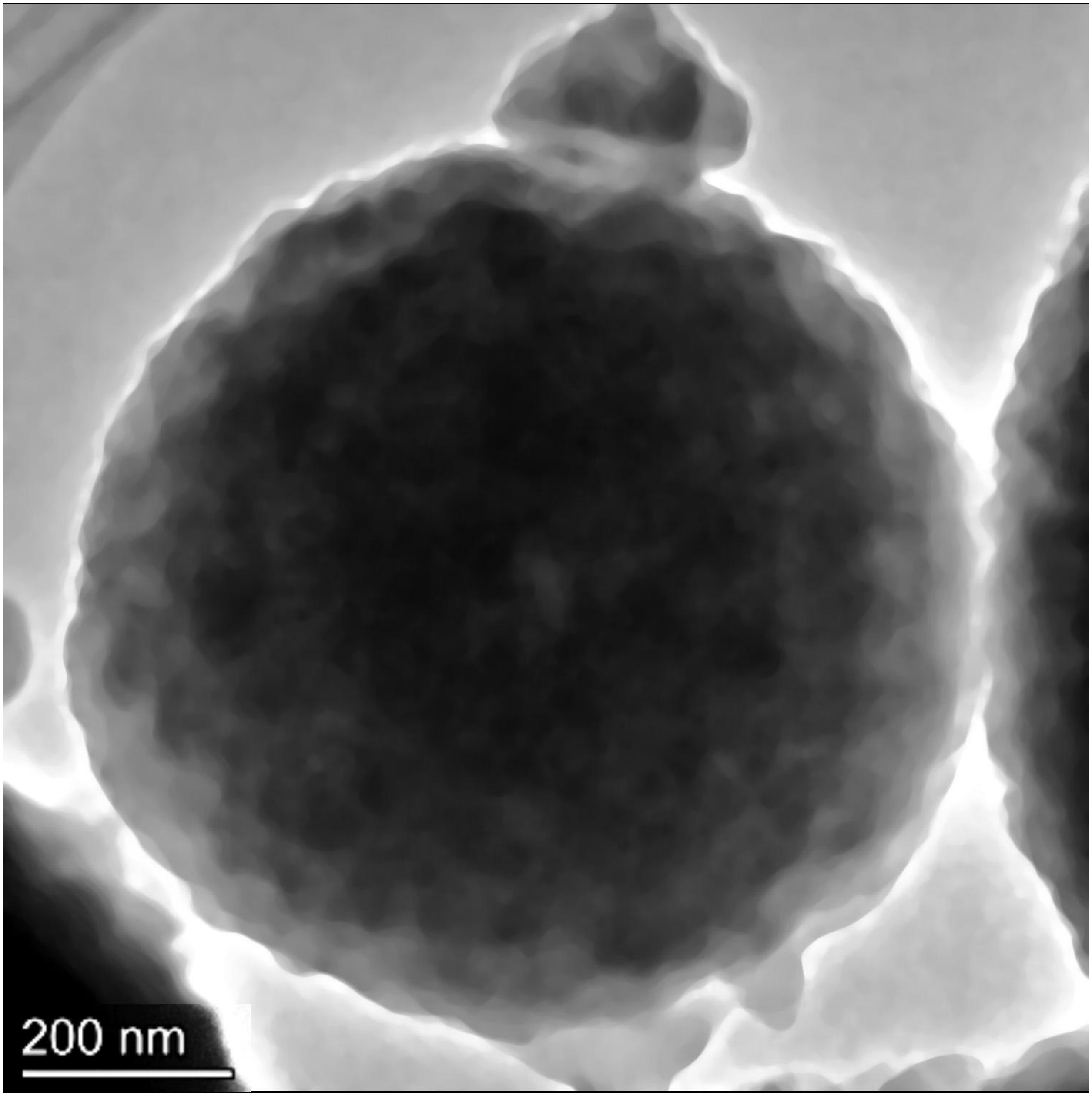

Figure S3. Bright-field TEM image of a single FSY HACC sphere. Same image as in Fig. 2c, though the image has been processed to better reveal the internal mottled structure.

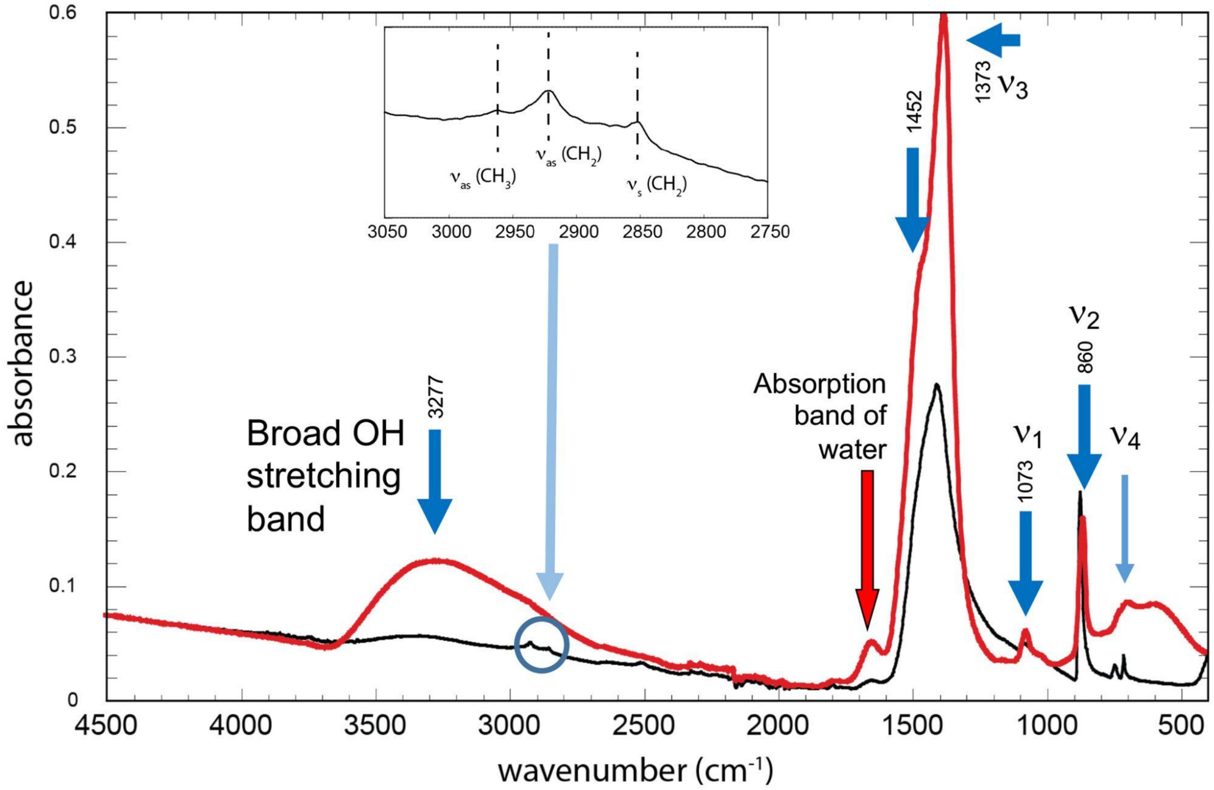

Figure S4. IR spectra of fresh FSW HACC (red spectrum) compared with peridial material that has weathered and crystallized in the desert (black spectrum). The mineralized specimen shows sharp ν_4 bands for calcite (712 cm^{-1}) and vaterite (744 cm^{-1}). The inset spectrum more clearly shows the organic matter absorption bands that sit on the broad OH stretching band.

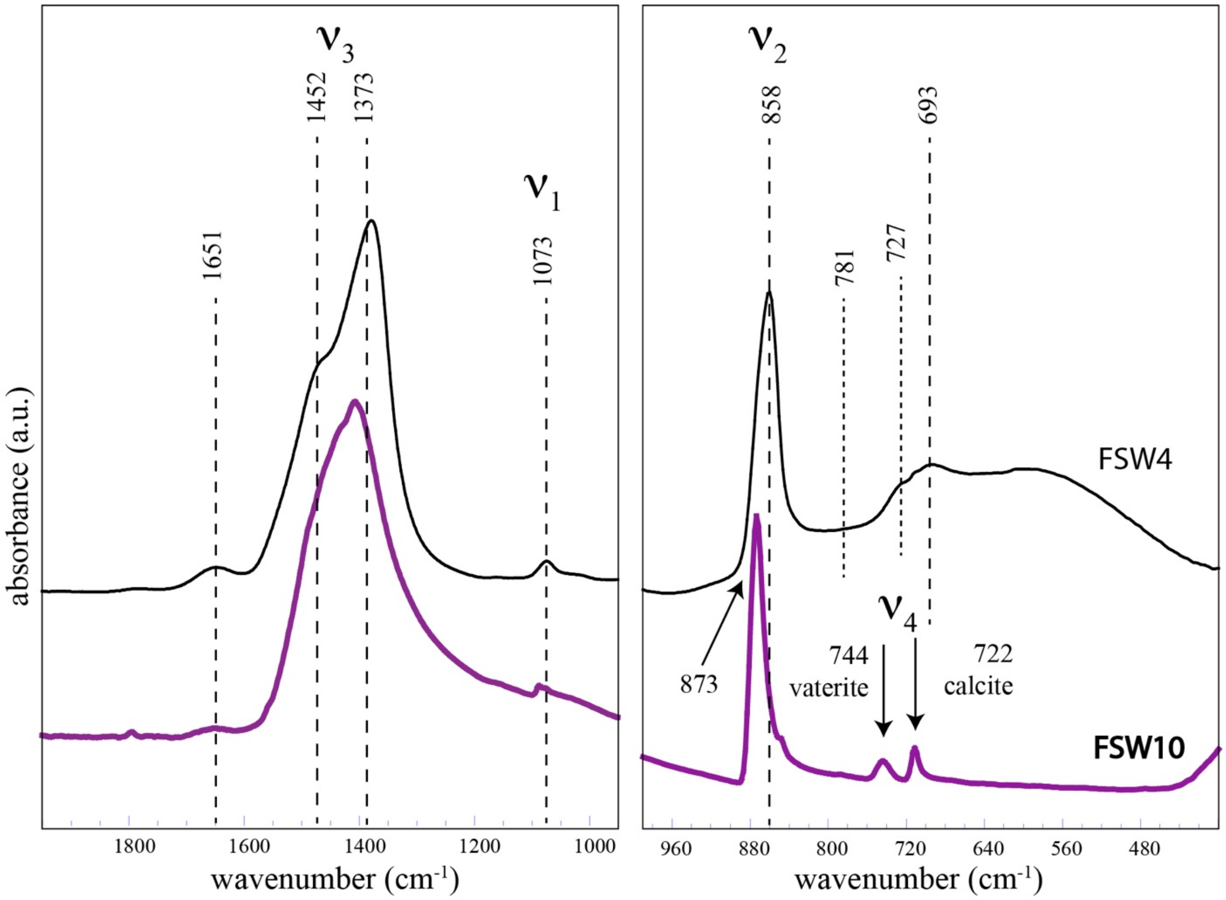

Figure S5. IR spectra of fresh FSW (sample FSW4) HACC compared with peridial material that has weathered and crystallized in the desert (FSW10). The mineralized specimen shows sharp ν_4 bands for calcite (712 cm^{-1}) and vaterite (744 cm^{-1}).

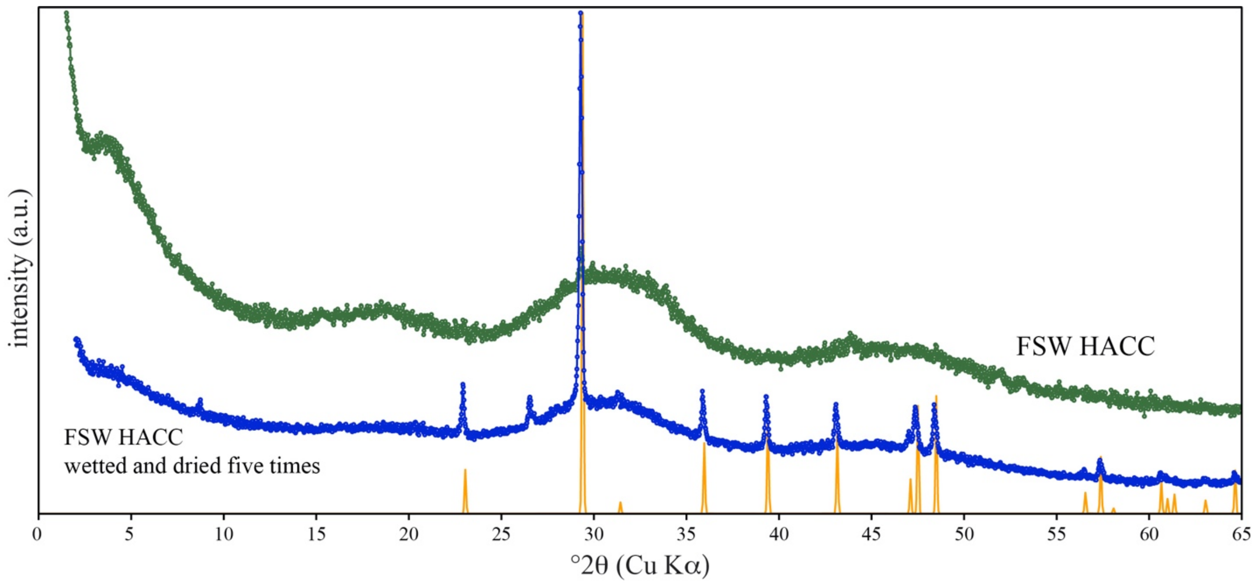

Figure S6. Powder x-ray diffraction pattern of fresh FSW HACC (as collected, green pattern) compared with the same sample after five sessions of wetting and drying (blue pattern). The simulated pattern for calcite (orange reflections) is shown at the bottom of the image. Patterns offset vertically for clarity.

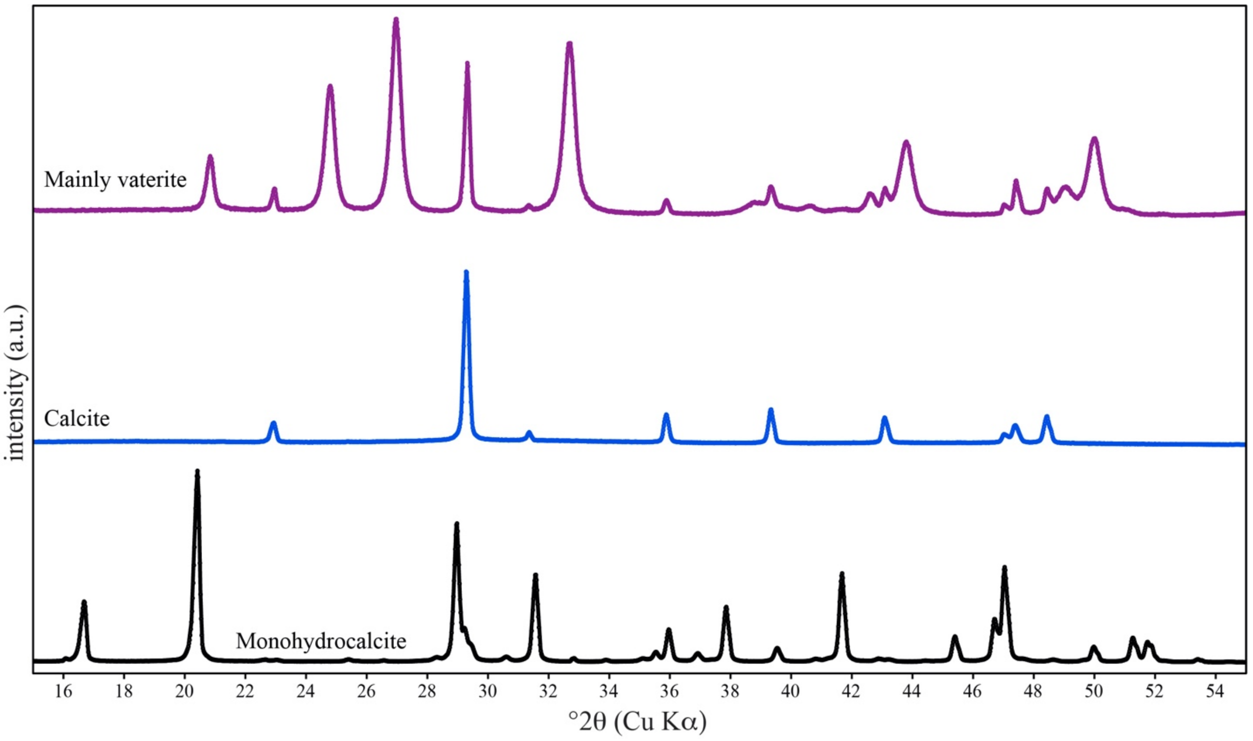

Figure S7. Powder x-ray diffraction patterns showing the range of minerals formed from weathering of the Arizona (FSW) *Fuligo septica* HACC in the desert. Patterns offset vertically for clarity.

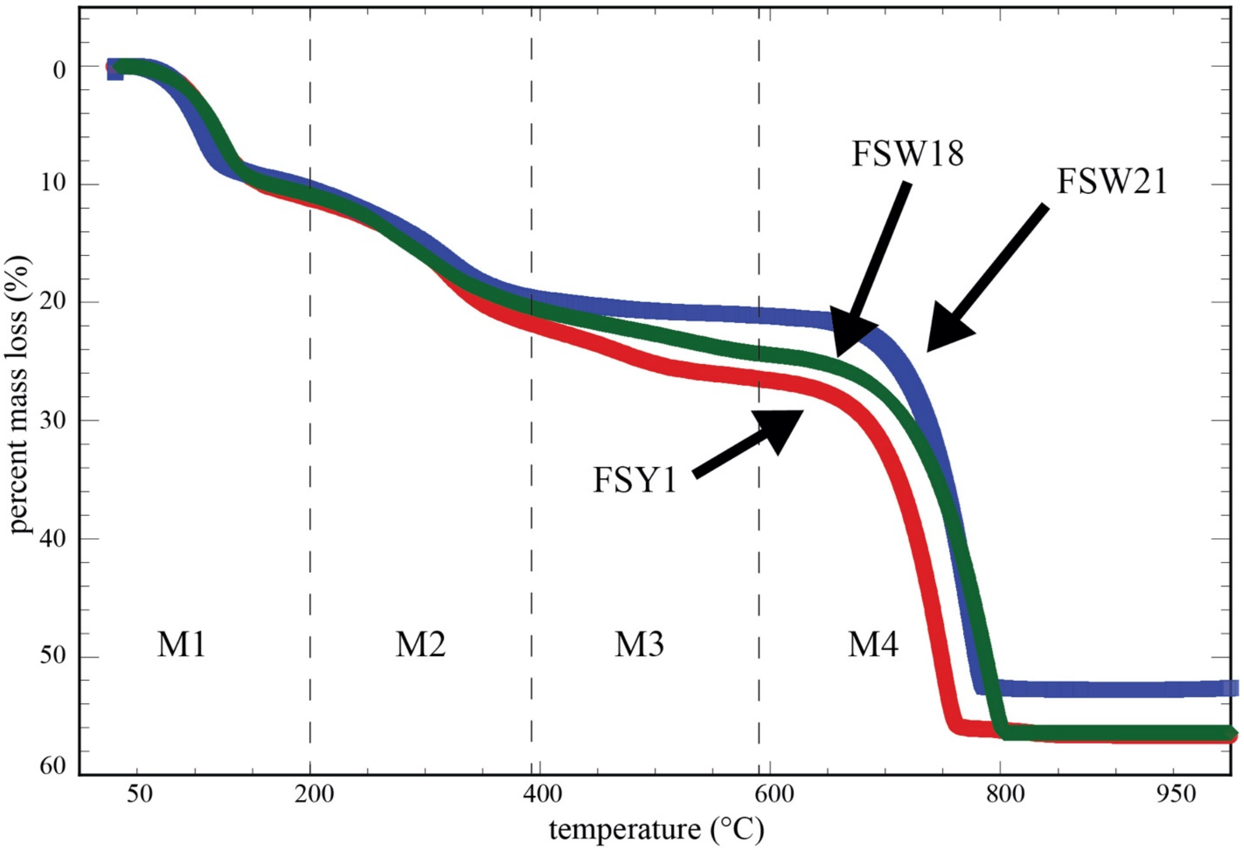

Figure S8. Thermogravimetric (TG) curves for two FSW HACC samples (FSW18 and 21) and FSY1 showing the consistent thermal behaviors for each. M1 to M4 refer to the four distinct mass-loss ranges discussed in the text.

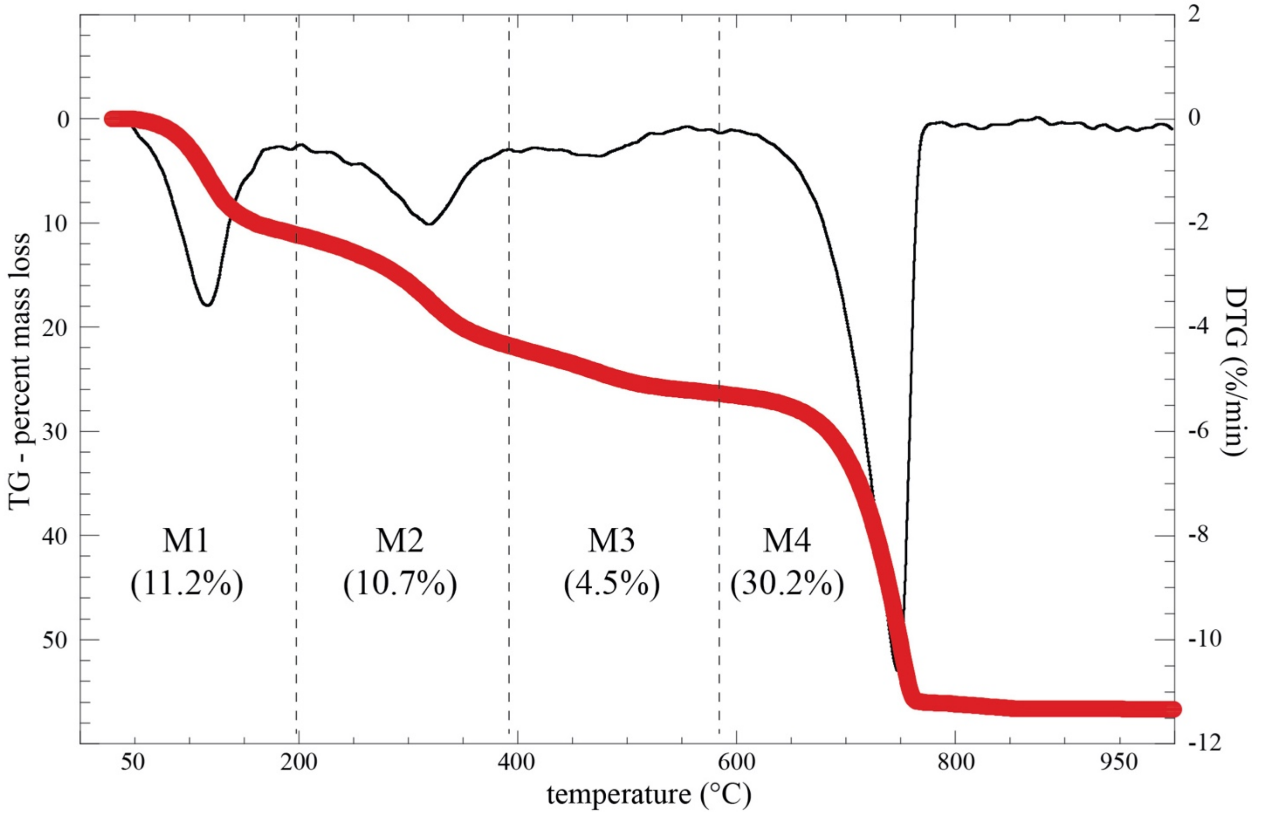

Figure S9. TG (thick red line) and DTG (thin black line) of the FSY HACC. M1 to M4 refer to the four distinct mass-loss ranges discussed in the text, and the corresponding percent mass loss for each range in parentheses. DTG – differential thermogravimetric analysis.

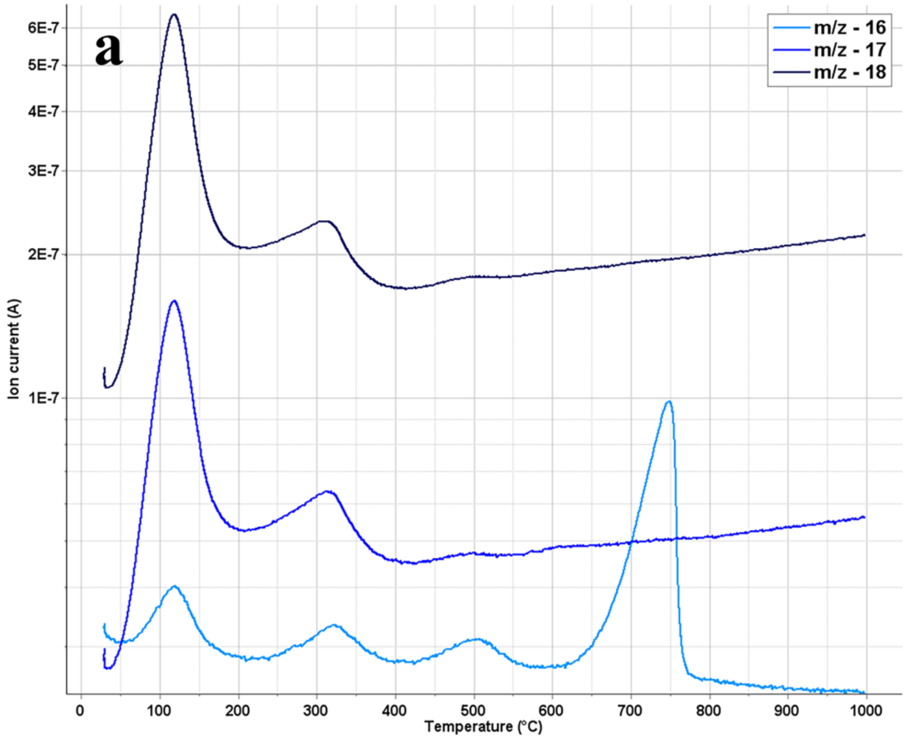
a
m/z - 16
m/z - 17
m/z - 18
6E-7
5E-7
4E-7
3E-7
2E-7
1E-7
Ion current (A)
0
100
200
300
400
500
600
700
800
900
1000
Temperature (°C)

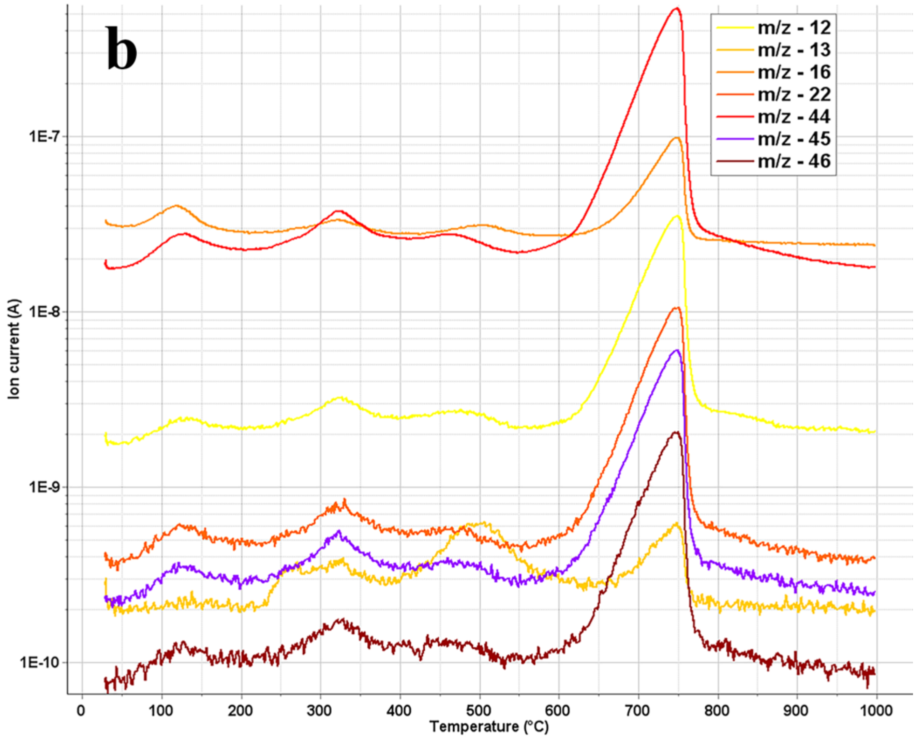
b
m/z - 12
m/z - 13
m/z - 16
m/z - 22
m/z - 44
m/z - 45
m/z - 46
1E-7
1E-8
1E-9
1E-10
Ion current (A)
0
100
200
300
400
500
600
700
800
900
1000
Temperature (°C)

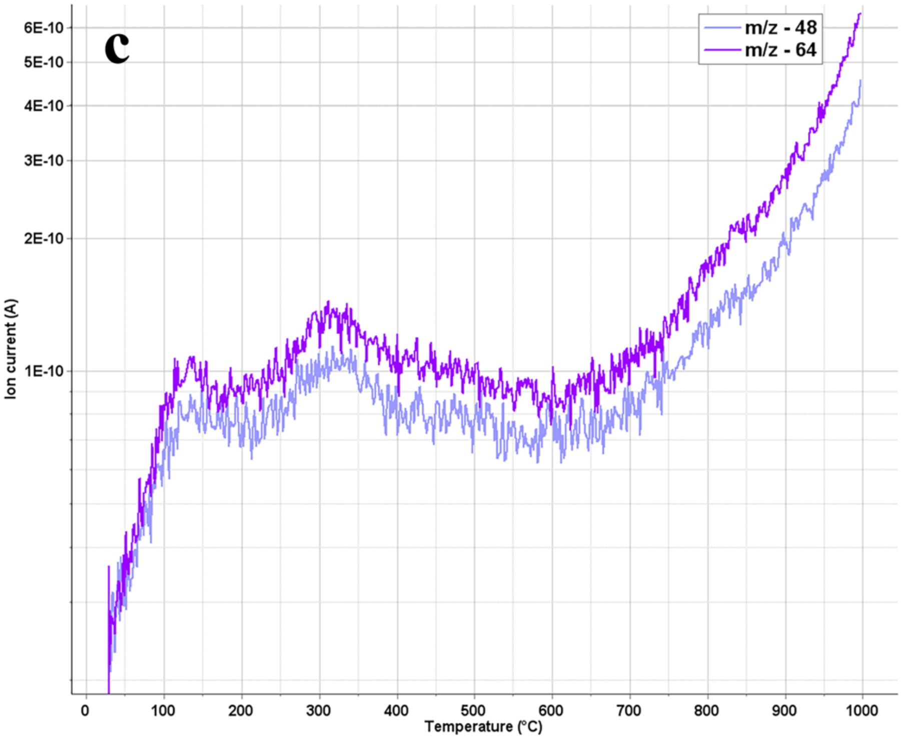
c
m/z - 48
m/z - 64
Ion current (A)
6E-10
5E-10
4E-10
3E-10
2E-10
1E-10
Temperature (°C)
0
100
200
300
400
500
600
700
800
900
1000

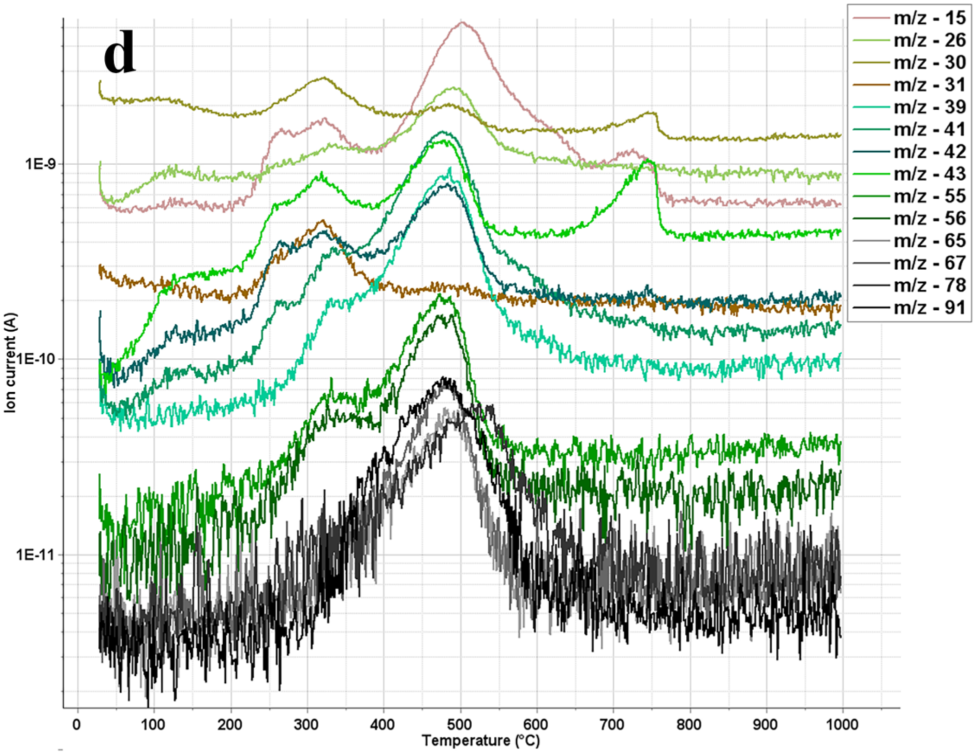
d
m/z - 15
m/z - 26
m/z - 30
m/z - 31
m/z - 39
m/z - 41
m/z - 42
m/z - 43
m/z - 55
m/z - 56
m/z - 65
m/z - 67
m/z - 78
m/z - 91
Ion current (A)
1E-9
1E-10
1E-11
Temperature (°C)
0
100
200
300
400
500
600
700
800
900
1000

Figure S10. Selected EGA profiles for the FSY ACC. **a**) Water (m/z=18) and OH^- (m/z=17), and O^- (m/z=16). **b**) Various singly and doubly charged CO_2, C, and isotopic (^{13}C, ^{17}O, ^{18}O, ^{16}O) bearing molecules. **c**) S-bearing species, m/z=48 for SO^+ and m/z=64 for S_2^+. **d**) Selected organic species, e.g., m/z=15 for CH_3^+, m/z=26 for $C_2H_2^+$, m/z=30 for CH_2O^+, and m/z=91 for the benzyl group $C_6H_5CH_2^-$. Additional assignments are listed in Table 7.

Table S7. Tentative formula assignments of the detected masses with possible sources, shown on figures S10a, b, c and d.

Fragment (m/z)	Possible formula (and source)
	Figure S10 a
16	O^+ (water)
17	OH^+, NH_2^+ (water, amines)
18	H_2O^+ (water)
	Figure S10 b
12	$^{12}C^+$ (organics, carbon dioxide)
13	$^{13}C^+$ (organics, carbon dioxide)
16	O^+ (water)
22	$^{12}CO_2^{++}$ (carbon dioxide)
44	$^{12}CO_2^+$ (carbon dioxide)
45	$^{13}CO_2^+$ (carbon dioxide)
46	$^{12}C^{18}O^{16}O_2^+$ (carbon dioxide)
	Figure S10 c
48	SO^+ (sulfates, sulfur containing organics)
64	SO_2^+, S_2^+ (sulfates, sulfur containing organics)
	Figure S10 d
15	CH_3^+ (methyl derivatives, alkyl groups)
26	$C_2H_2^+$ (aromatic hydrocarbons)
30	CH_2O^+, $NH_2CH_2^+$, $C_2H_6^+$ (methyl ethers, alkyl groups)
31	CH_3O^+ (methoxy derivatives, methyl esters)
39	$C_3H_3^+$ (aromatic hydrocarbons?)
41	$C_3H_5^+$ (propyl esters?)
42	CH_2CO^+, $C_3H_6^+$, $C_2H_4N^+$ (acetates, *N*-acetyl compounds, butyl ketones)
43	CH_3CO^+, $C_3H_7^+$, $HNCO^+$ (propyl derivatives, methyl ketones, alkanes)
55	$C_4H_7^+$ (butyl esters?)
56	$C_4H_8^+$ (polyunsaturated hydrocarbon chain)
65	$C_5H_5^+$ (polyunsaturated hydrocarbon chain)
67	$C_5H_7^+$ (polyunsaturated hydrocarbon chain)
78	$C_6H_6^+$, (aromatic hydrocarbons?, polyunsaturated hydrocarbon chain)
91	$C_7H_7^+$ (aromatic hydrocarbons?, polyunsaturated hydrocarbon chain)

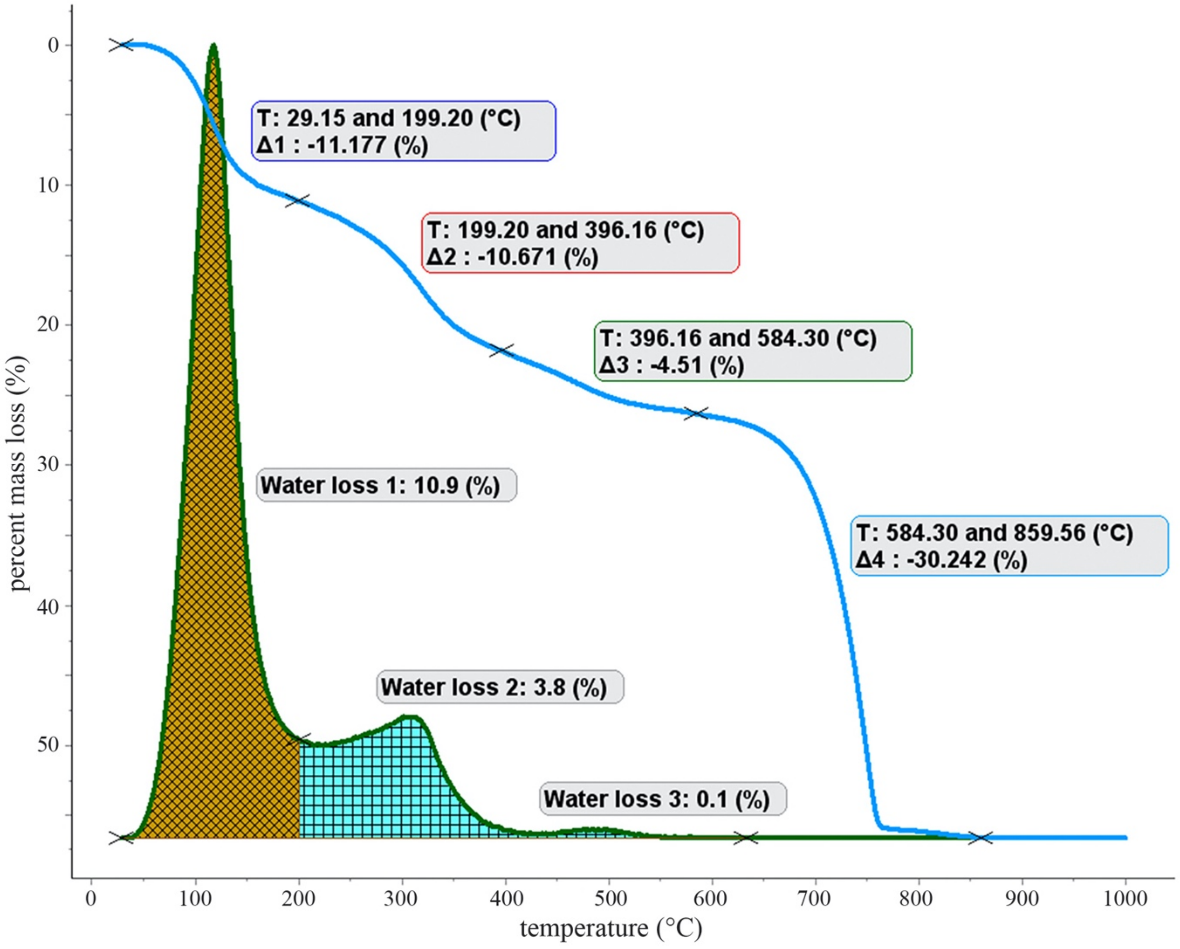

Figure S11. Comparison of the TG curve (blue curve) for the FSY HACC and the quantified water content as measured by EGA with respect to temperature. See **Water Calculation from TG-DSC-MSEGA measurements** above for details of the water calculation.

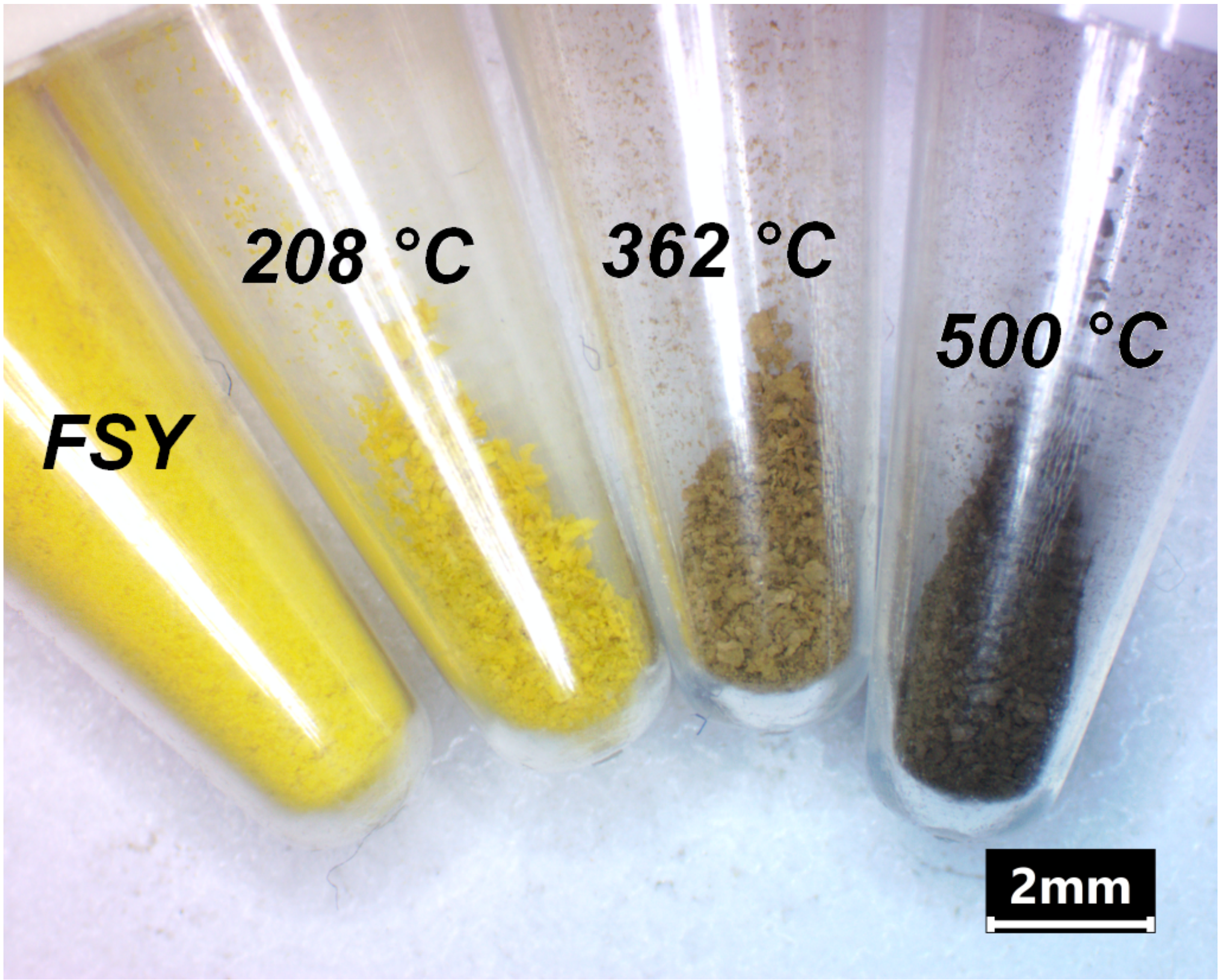

Figure S12. Color of FSY room temperature HACC (far left) and after heating at various temperatures in a He atmosphere. The changes in color are the result of gradual degradation of the organic matter.

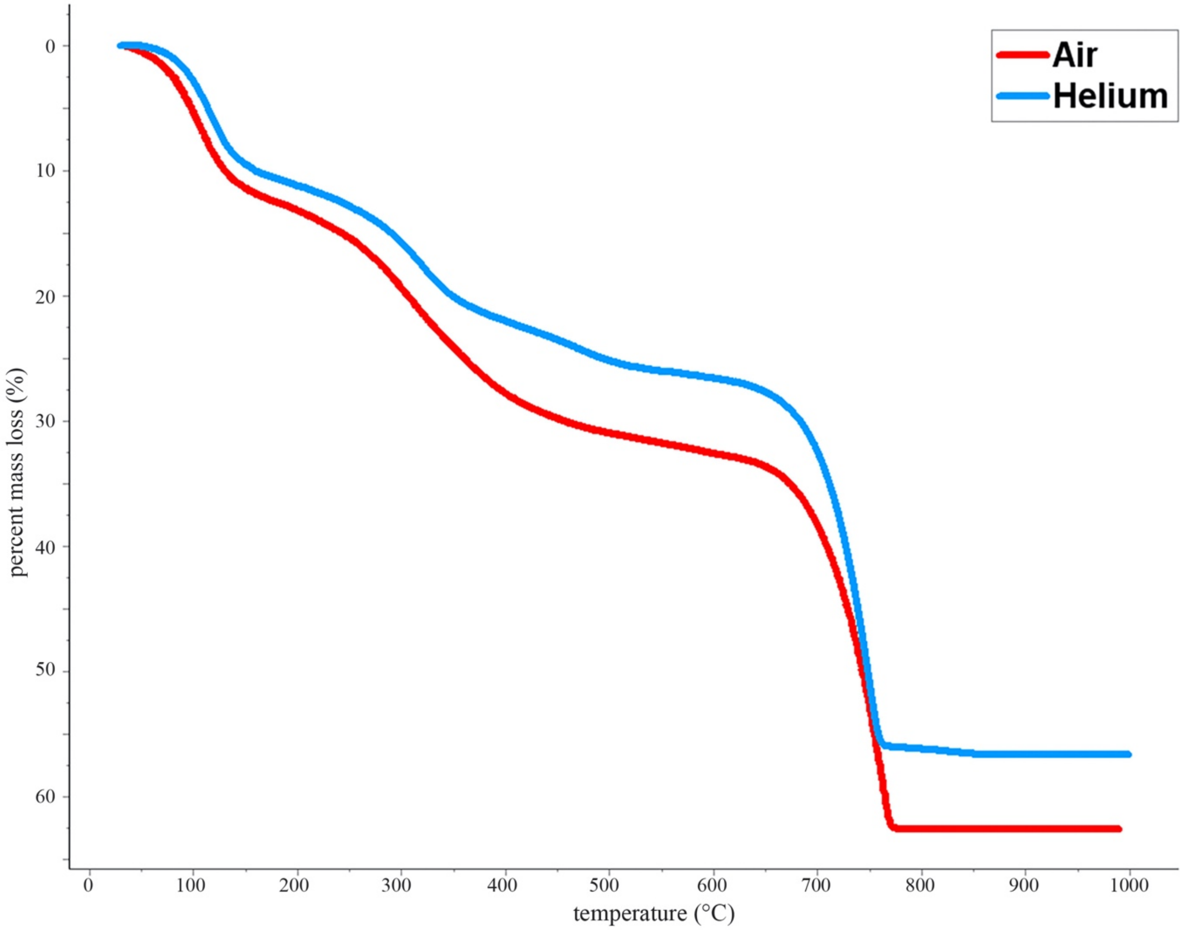

Figure S13. Comparison of the TG curve for the FSY HACC run under helium (upper curve, blue) and air (lower curve, red).

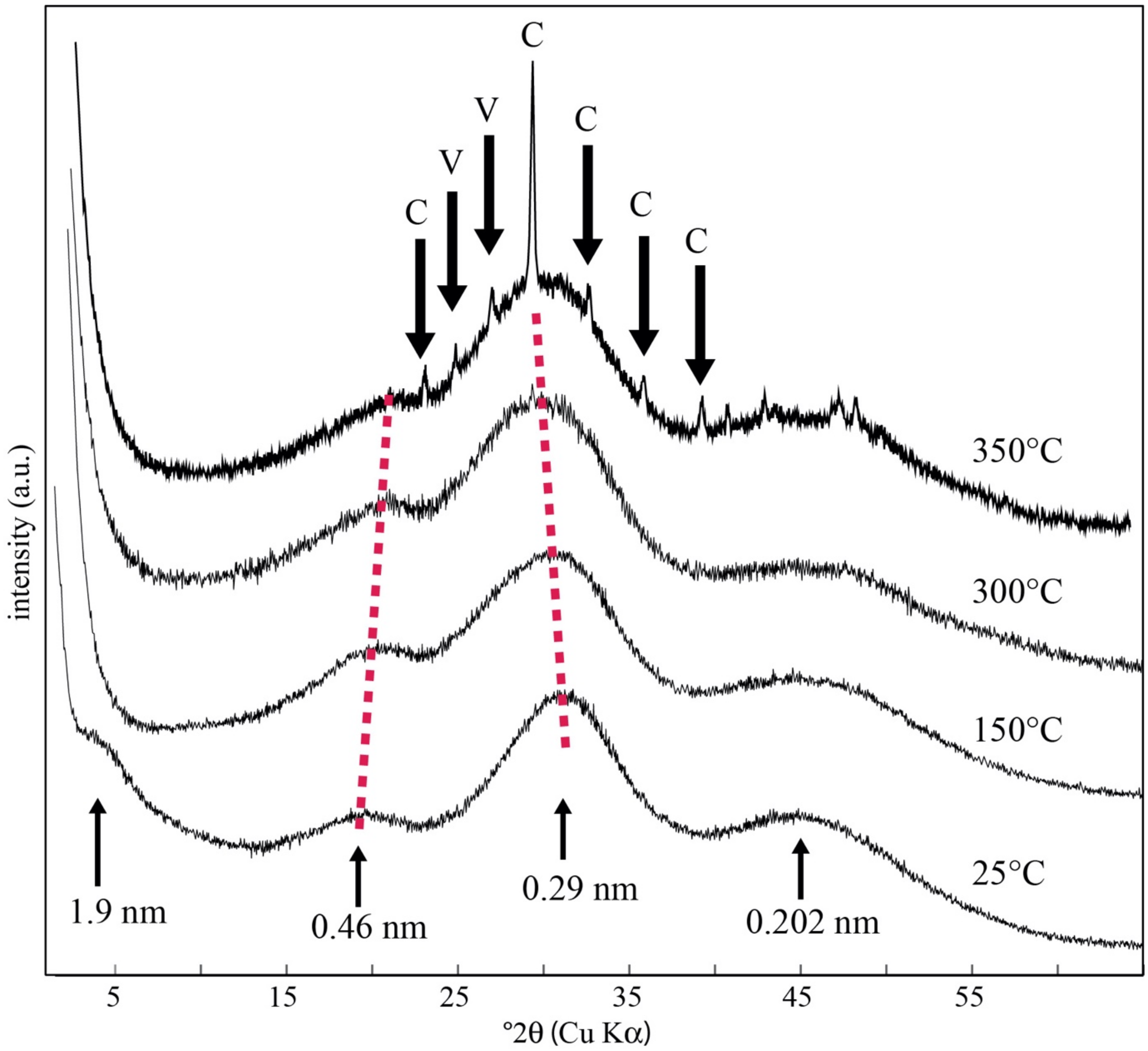

Figure S14. Powder x-ray diffraction pattern of the as-collected FSY peridium HACC (25 °C pattern), and the patterns after heating in air to 150, 300, and 350 °C. c- calcite. v- vaterite. The dotted line shows the shift of the 0.46 and 0.29 nm reflections with respect to temperature. Patterns shifted vertically for clarity.

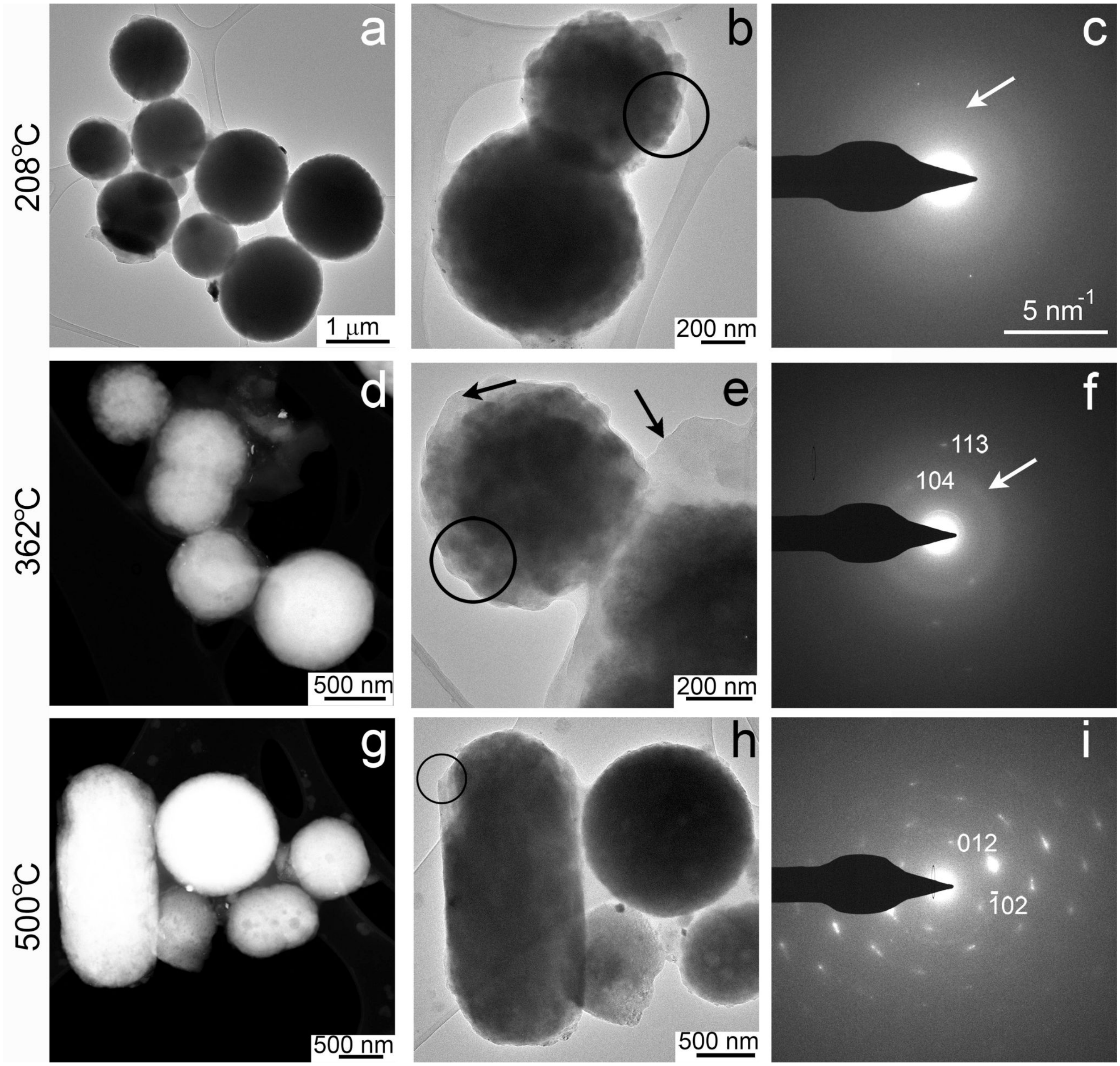

Figure S15. Morphology and structure of the *Fuligo septica* peridial HACC after heating. (**a**) BFTEM images of a precipitate heated at 208 °C. (**b**) BFTEM image of selected spheres. (**c**) SAED pattern acquired from the circled area of **b**). (**d**) HAADF-STEM image of a precipitate heated at 362 °C. (**e**) BFTEM image of a selected sphere surrounded by low contrast material (black arrows). (**f**) SAED pattern (acquired from the circled area of **e**) with calcite indices and a diffuse ring (white arrow). (**g**) and (**h**) HAADF-STEM and BFTEM image of the precipitate heated at 500 °C. The spherical morphology is dominant but elongated grains also occur. (**i**) SAED pattern acquired from the black circled area of (**h**) shows diffraction spots of calcite.

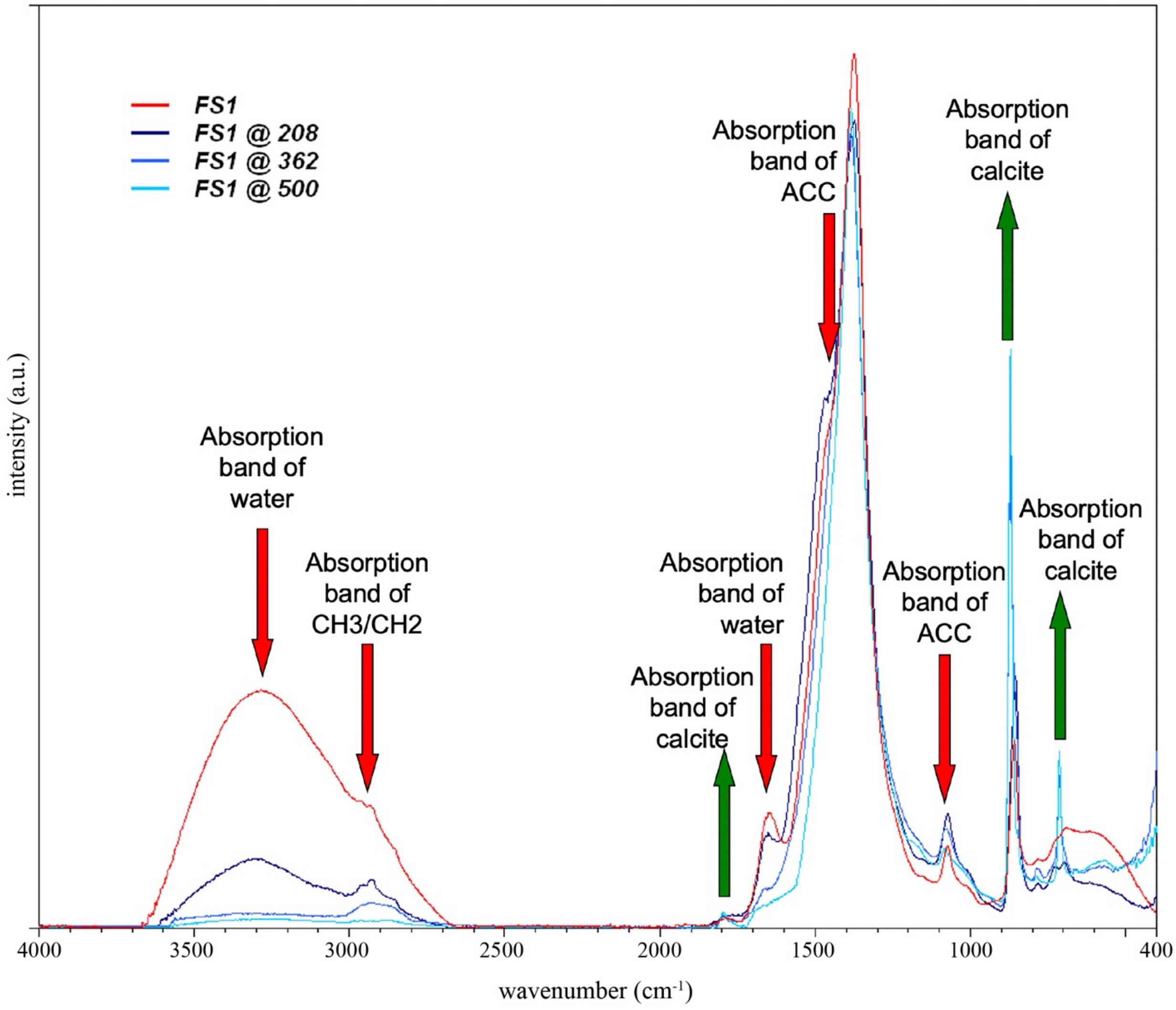

Figure S16. IR absorption spectra of the FSY peridial HACC acquired at room temperature (red curve), compared with samples heated to 208, 362, and 500 °C.

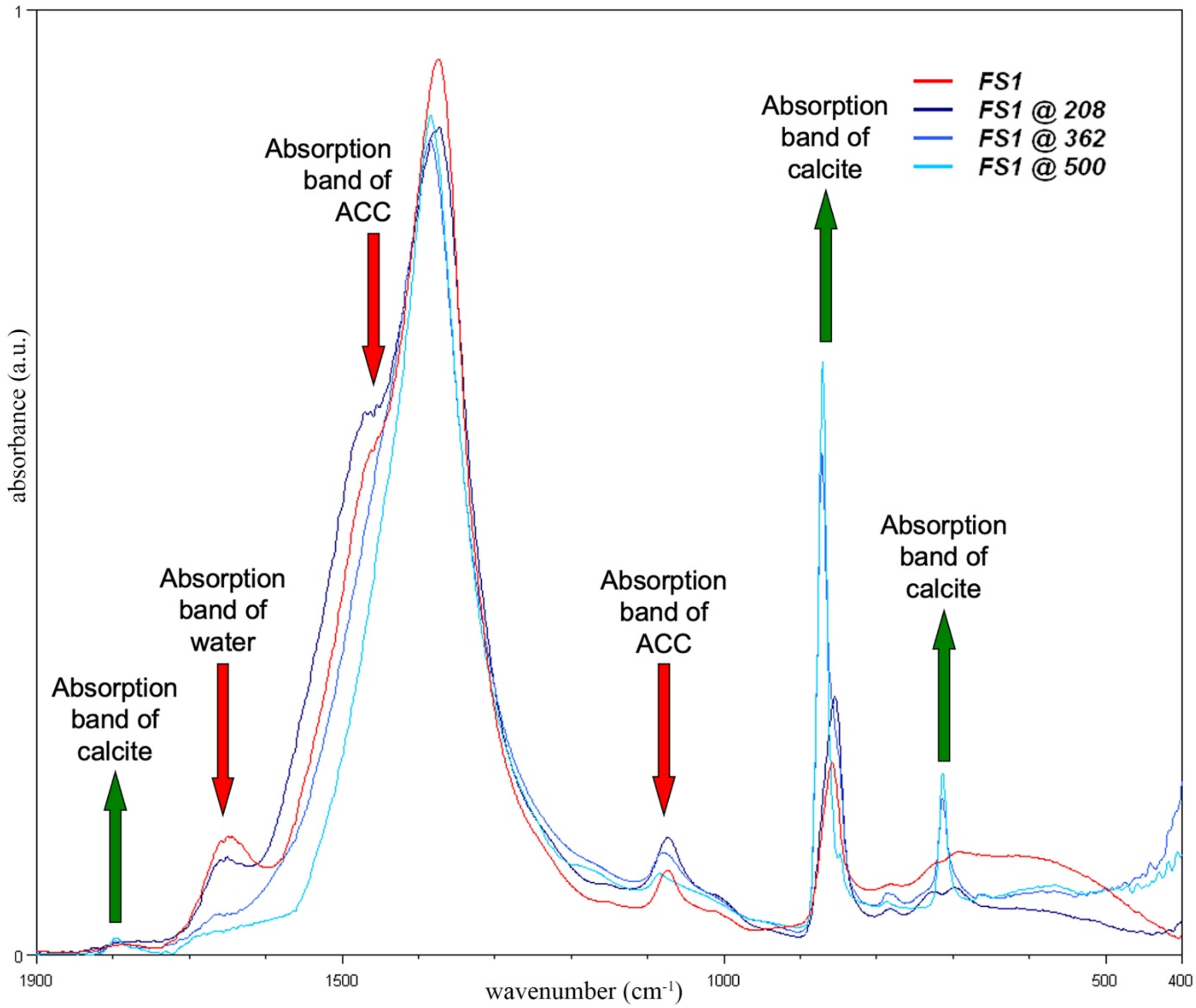

Figure S17. IR absorption spectra of the FSY peridial HACC acquired at room temperature (red curve), compared with samples heated to 208, 362, and 500 °C, showing the ν_4 and ν_2 absorption bands for calcite appearing at 362 °C.

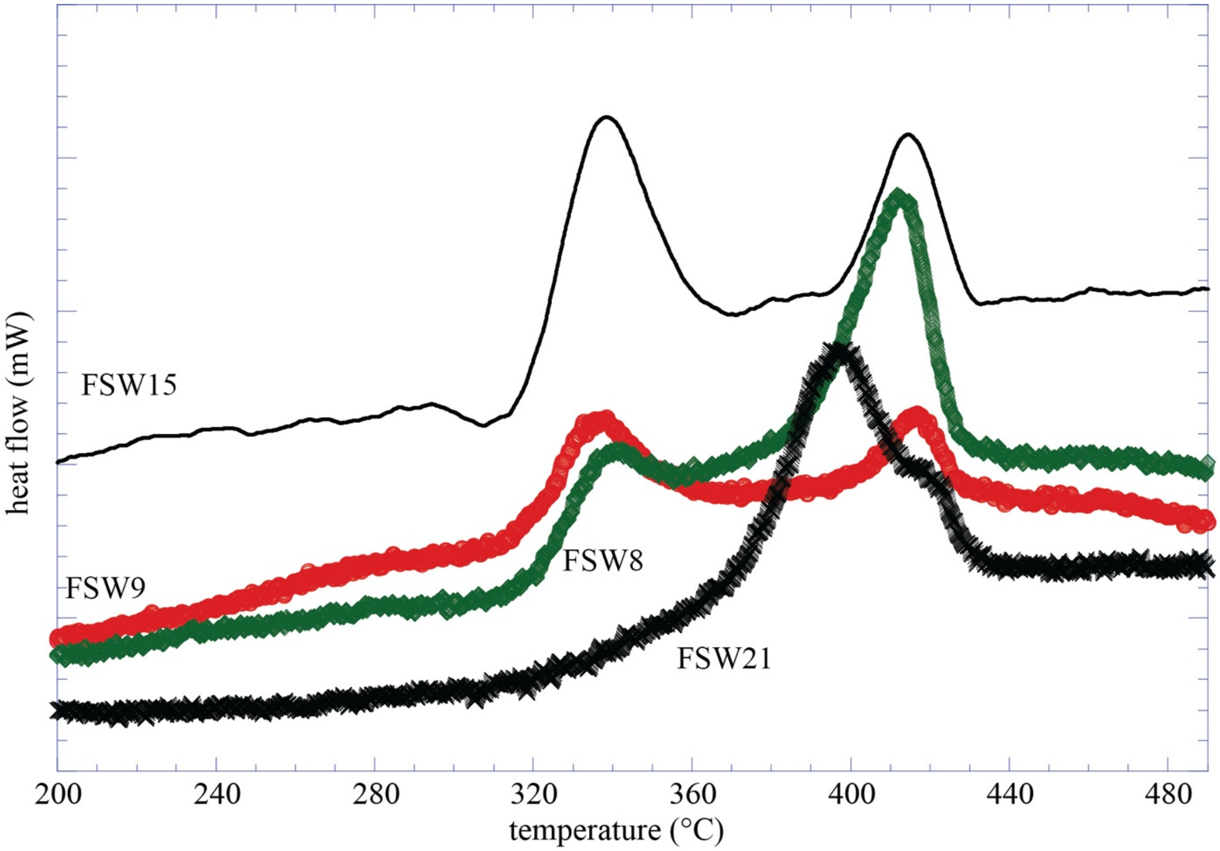

Figure S18. The exothermic DSC peaks for four selected FSW samples.

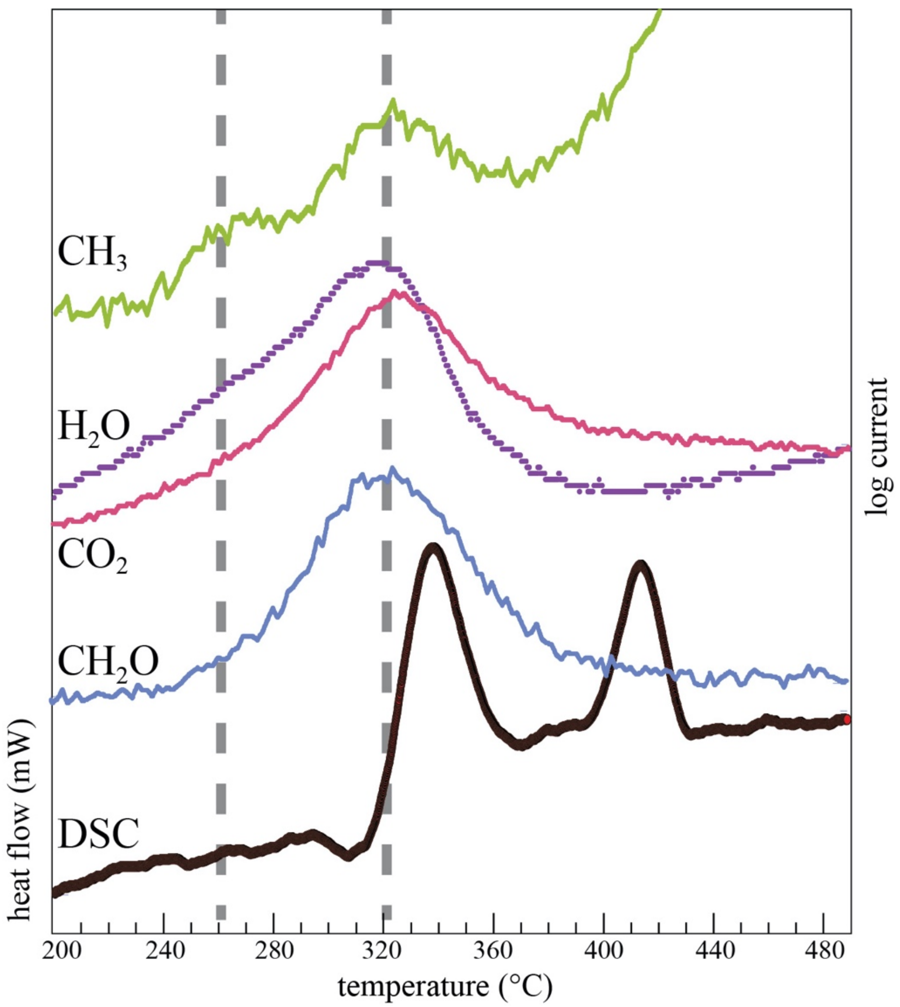

Figure S19. Comparison of the exothermic DSC region (in mW) for the FSW (FSW15, Fig. S17) sample run under He (black curve at bottom), compared with selected EGA profiles (log current) corresponding to CH_2O^+ (m/z=30), $^{12}CO_2^+$ (m/z=44), H_2O^+ (m/z=18), and CH_3^+ (m/z=15). Additional gas assignments are listed in Table S7. All profiles are scaled and shifted along the y-axis so as to show the correspondence between the peak maxima. The absolute EGA scales are shown in Fig. S10.

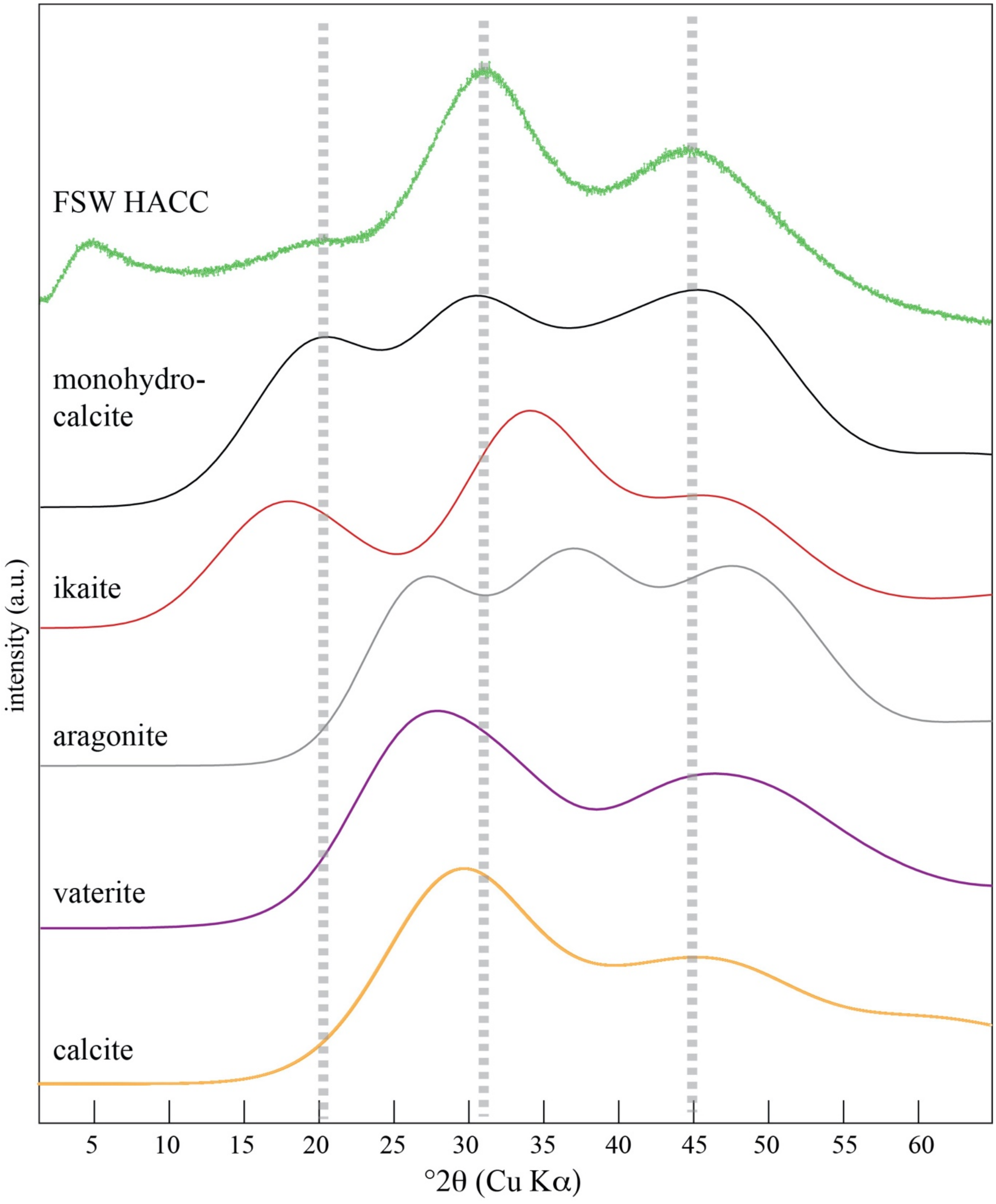

Figure S20. Experimental powder x-ray diffraction pattern for the FSW HACC compared with the simulated patterns for the hydrated and anhydrous $CaCO_3$ polymorphs. The simulations are performed for a scattering grain-size of 1 nm.

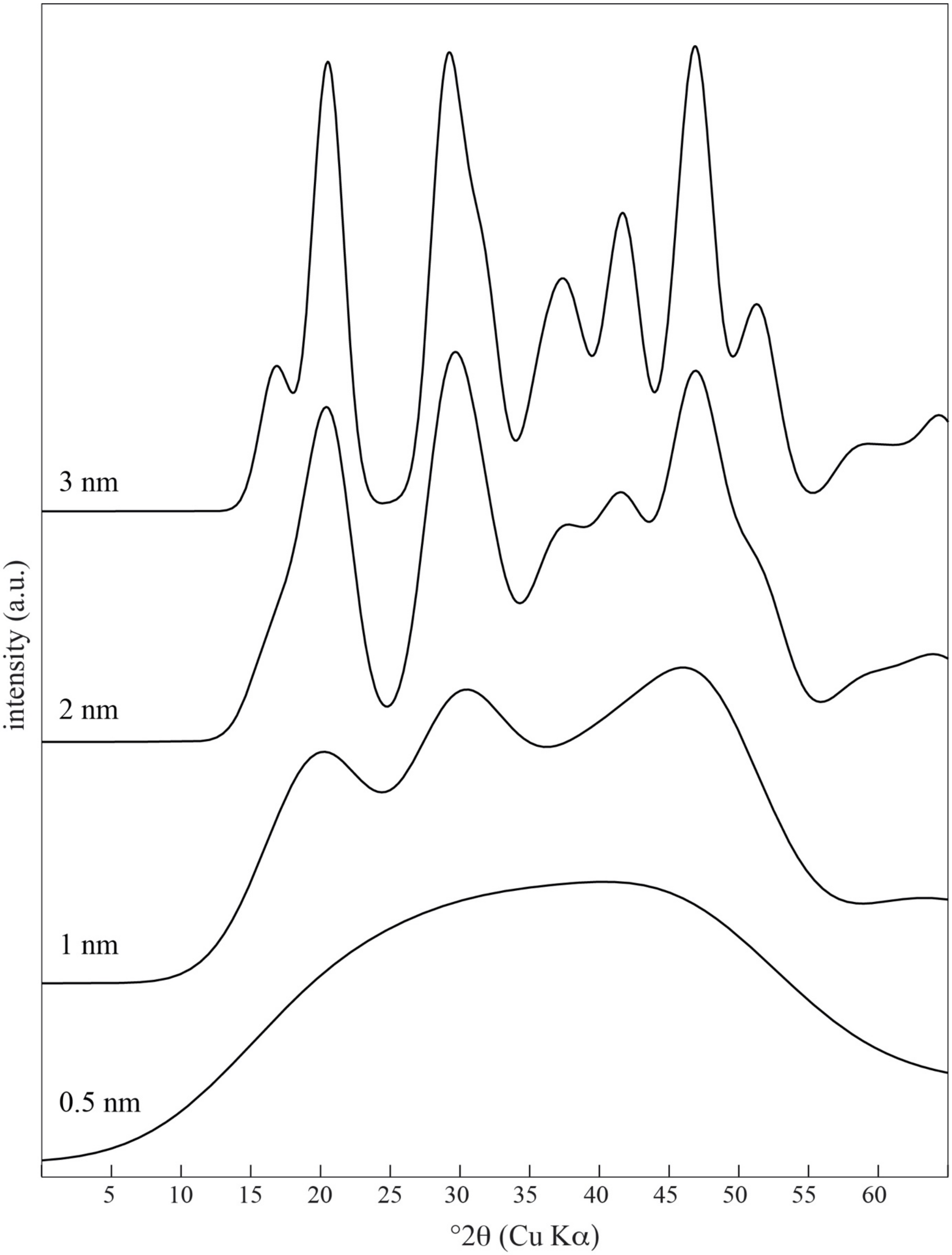

Figure S21. Simulation of the x-ray diffraction profile of monohydrocalcite with respect to diffracting grain size.
